# Long noncoding RNA, *PURPL* is associated with aneuploidy and its magnitude of expression level is dependent on P53 status

**DOI:** 10.3389/fcell.2024.1410308

**Published:** 2024-12-23

**Authors:** Pushkar Malakar

**Affiliations:** Department of Medical Biotechnology, School of Biological Sciences, Ramakrishna Mission Vivekananda Educational and Research Institute (RKMVERI), Kolkata, India

**Keywords:** long noncoding RNA, PURPL, chromosomal instability, p53, multinucleation, altered nuclear shape, and aneuploidy

## Abstract

**Introduction:**

Long non-coding RNAs (lncRNAs) are a fascinating, but still largely uncharacterized, class of genes. Recently, lncRNAs have attracted significant attention due to their emerging functions in development and disease. The role of lncRNAs in chromosome instability or aneuploidy is not extensively studied.

**Methods:**

We started with the objective of characterizing lncRNAs that play an important role in chromosome instability (CIN) or aneuploidy. Here, we report the initial functional characterization of PURPL in the context of chromosomal instability or aneuploidy.

**Results:**

We report the over-expression of lncRNA PURPL in three experimental models of chromosomal instability, or aneuploidy. In addition, the study also showed that the extent or magnitude of PURPL expression is dependent upon p53 status. Our research also showed that turning off PURPL is enough to create a CIN phenotype in RPE-1 cell lines that were previously karyotypically stable. Moreover, PURPL knockdown cells are more sensitive to CIN or aneuploidy inducers.

**Discussion:**

These findings show that PURPL is essential for maintaining chromosomal or genomic stability in mammalian cells. Collectively, the study demonstrated that lncRNA-PURPL significantly contributes to CIN, or aneuploidy.

## Introduction

Long noncoding RNAs, or lncRNAs, have recently attracted significant attention due to their emerging functions in development and disease (Schmitz et al., 2016). lncRNAs belong to the diversified family of RNAs that are >200 nucleotides in size and lack any detectable open reading frame (ORF) (Lee et al., 2016). The exact number of lncRNAs encoded in the human genome is not known precisely, but most studies place it around tens of thousands (Guttman et al., 2009). The biological roles and molecular functions of the majority of lncRNAs remain to be investigated (Lee et al., 2016). Studies have shown that lncRNAs in the nucleus regulate transcription in cis or trans, organize subnuclear structure, and mediate chromosomal interactions (Long et al., 2017). Nevertheless, studies of lncRNA function are still at an early stage (Noh et al., 2018).

Maintenance of genome integrity is of utmost importance for organism survival and for the inheritance of traits to offspring (Papamichos-Chronakis and Peterson, 2012). Genomic instability arises from DNA damage, aberrant DNA replication, or uncoordinated cell division, leading to chromosomal aberrations and gene mutations (Papamichos-Chronakis and Peterson, 2012). Higher-order chromatin structures package eukaryotic genomes and ultimately organize them in a manner that functionally relates to gene expression (Van Bortle and Corces, 2012). Understanding the mechanisms and molecular players involved in genome organization will help to decipher the role played by genome organization in gene expression (Van Bortle and Corces, 2012). The role of lncRNA in genomic organization is not known. Studies have demonstrated the crucial role of P53 in preserving the integrity of the genome (Pitolli et al., 2019).

More than 50% of human cancers contain mutations in the tumor suppressor p53 (Ozaki and Nakagawara, 2011). When cells are under stress, like when DNA is damaged, p53 directly starts the transcription of many genes that code for proteins. These genes control many cellular processes, such as cell cycle arrest, apoptosis, and senescence (Bieging et al., 2014). p53 was shown to regulate the expression of several lncRNAs, including lincRNA-p21, PANDA, DINO, PURPL, etc. (Li et al., 2017). RNA sequencing (RNA-seq) identified PURPL (p53 upregulated regulator of p53 levels) as an intergenic lncRNA from multiple colorectal cancer (CRC) lines (Li et al., 2017). Loss of PURPL resulted in elevated basal p53 levels and impaired cell growth *in vitro* and *in vivo* (Li et al., 2017). The lncRNA PURPL was one of the transcripts most highly and consistently elevated among senescent cells (Casella et al., 2019). Recent studies have shown that the transcription factor p53, generally elevated in senescence conditions, transcriptionally regulates PURPL production (Casella et al., 2019). In addition, PURPL may help senescent cells stay alive by acting as a pro-survival factor in these cells, which is known to make them more likely to become tumors (Casella et al., 2019). It was shown that PURPL could be a more robust marker transcript for senescence than p16 mRNA or p21 mRNA, as the latter two mRNAs did not reach significance cut-offs in all senescence models, as determined by RNA-seq and qRT-PCR analyses (Casella et al., 2019).

Senescence is associated with aneuploid cells or near-polyploid cells having an abnormal number of chromosomes or chromosomal instability (He et al., 2018). Chromosomal instability (CIN) is a type of genomic instability in which chromosomes are unstable, such that either entire chromosomes or sections of chromosomes are duplicated or deleted (Vargas-Rondón et al., 2017). In addition, CIN refers more specifically to the increase in the rate of addition or loss of whole chromosomes or parts of them (Thompson et al., 2010). CIN will lead to unequal distribution of DNA to daughter cells during mitosis, resulting in failure to maintain the correct number of chromosomes (euploidy), culminating in aneuploidy (incorrect number of chromosomes) (Potapova et al., 2013). CIN is the most common type of genomic instability and is considered to be an important cause of aneuploidy (Vargas-Rondón et al., 2017). CIN has been studied in solid tumors, and it was discovered that CIN is a common feature in solid and hematological cancers, especially colorectal cancer (Vargas-Rondón et al., 2017).

The presence of micronuclei is a hallmark of chromosome instability (Crasta et al., 2012). Micronuclei are formed when one or a few chromosomes do not join to form a daughter nucleus but instead form their own nuclear envelope (Crasta et al., 2012). The interaction between double-stranded DNA (dsDNA) from micronuclei and the cytoplasm during interphase triggers the activation of the cGAS-STING (cyclic GMP-AMP synthase-Stimulator of Interferon Genes) pathway (Bakhoum and Cantley, 2018). The prolonged activation of the cGAS-STING pathway generated by CIN leads to the reconfiguration of downstream signals in cancer cells, resulting in the development of a tumor microenvironment that promotes metastasis or resistance to therapy (Bakhoum and Cantley, 2018).

There is a close relationship between abnormal nuclear shape and chromosomal instability (Gisselsson et al., 2001; Pons et al., 2022). A linear and positive correlation was observed between the abnormal nuclear shape on the one hand and cells with chromosomal instability features like unstable chromosomes and anaphase bridges on the other hand (Gisselsson et al., 2001). This correlation between abnormal nuclear shape and chromosomal instability was also applicable in tumors (Gisselsson et al., 2001). Abnormalities in nuclear shape are regarded as an indicator of chromosomal instability and genetic instability (Van Bortle and Corces, 2012; De las Heras et al., 2013; Denais and Lammerding, 2014). Cell multinucleation is closely associated with chromosomal instability (He et al., 2018; Pantelias et al., 2019; Dhital et al., 2023). Polyploidy cells were shown to have defective chromosome segregation, resulting in aneuploidy (Potapova et al., 2013). Furthermore, polyploidy is also associated with high levels of chromosome instability (Mayer and Aguilera, 1990). Studies have also shown that, as a result of CIN, cell cycle deregulation, or asynchronous cell cycle, occurs (Nano et al., 2019; Gemble and Basto, 2020; Hosea et al., 2024).

The role of lncRNAs in CIN or in maintaining genomic integrity is not studied extensively except for NORAD (Lee et al., 2016). The lncRNA NORAD was shown to be modulated in response to DNA damage. Furthermore, it was shown to play an important role in maintaining genome integrity by regulating the activity of the RNA-binding proteins PUM2 and PUM1 (Lee et al., 2016). A higher extent of aneuploid and near-polyploid cells in a given population led to senescence (He et al., 2018). Furthermore, chromosome missegregation generates cell cycle-assigned cells, or senescence cells (Santaguida et al., 2017). We were intrigued whether PURPL, which was shown to be upregulated in colorectal cancer, which is highly aneuploid, had anything to do with aneuploidy. Additionally, CIN was shown to induce senescence, a state where PURPL was highly upregulated, leading us to investigate the role of PURPL in CIN.

In this study, we investigated the regulation and function of the lncRNA PURPL in CIN, or genomic instability. We found increased PURPL expression is associated with chromosomal instability or genomic instability induced by reversine, cytochalasin-B, and an Aurora kinase inhibitor (ZM447439). PURPL overexpression upon induction of CIN or genomic instability is not solely dependent upon p53. In addition, we found that the extent of PURPL induction in CIN or genomic instability conditions is dependent on p53. Furthermore, knockdown of PURPL resulted in the CIN phenotype as well as in the deformed nucleus, along with a concomitant increase in the expression of MDM2. The study also found that PURPL knockdown cells are more sensitive to CIN or aneuploidy inducers. Our studies indicate that PURPL overexpression could be a more robust marker transcript for CIN or aneuploidy, as determined by qRT-PCR analyses in the models of chromosomal instability and aneuploidy tested. Altogether, our study provides functional insights on PURPL, demonstrating the role of this lncRNA in regulating genome integrity.

## Materials and methods

### Cell culture

RPE-1 cells were obtained from Arshad Desai Lab (UCSD). DLD1 cells were obtained from ATCC. MDA-MB-231 and HEK293T cells were obtained from NCCS, Pune. RPE-1 cells were grown in DMEM-F12 (Gibco-11330–032), DLD1 cells in RPMI (12633–012), MDA-MB-231 and HEK293T in DMEM (Himedia-AL007S) supplemented with 10% FCS (Thermo Fisher Scientific, A3160401), 0.1 mg/mL Penicillin Streptomycin (Gibco, 15140–122), 0.25 µg/mL Amphotericin B (Sigma, A2942), with (2 mM) L-Glutamine (Gibco, 25030–08). The inhibitors Cytochalasin-B (Abcam#ab143482), Aurora Kinase Inhibitor (Sigma#189410), and Riversine (Sigma#R3904) were dissolved in DMSO and used at the concentrations specified in the result section.

### Antisense oligonucleotides

All Antisense Oligonucleotides (ASO) transfections were performed for 72 h using Lipofectamine RNAi Max reagent (Thermo Fisher Scientific, 13778075) as per manufacturer’s instructions. For RNAi, ASO were diluted in Opti-MEM (Thermo Fisher Scientific, 31985088). ASO against PURPL (5′-/52MOErG/*/i2MOErA/*/i2MOErA/*/i2MOErG/*/) from IDT and control ASO (5′-/52MOErT/*/i2MOErT/*/i2MOErC/*/i2MOErT/*/) from IDT were used at a final concentration of 100 nM.

### Immunoblotting

Cell lysates were prepared using RIPA lysis buffer. Primary antibodies used were p21 (1:1,000; Cell Signaling Technology, 12D1), GAPDH (1:5,000; Invitrogen, MA5-15738) and p53 (1:1,000; Santa Cruz Biotechnology, SC-126Secondary antibodies (1:5,000 dilutions) were HRP-conjugated against rabbit (Cyvita, NA934V) and mouse (Cyvita, NA931V).

### Immunostaining/DAPI staining

For immunostaining, cells were fixed with ice-cold methanol for 5 min. Fixation was followed by blocking with 1% BSA in PBS/0.1% Tween (PBST) for 30 min at room temperature. Coverslips with cells were incubated in DAPI for 1 h and then washed 3 times in PBST. Cells were then mounted on slides using Prolong gold antifade mountant (Thermofisher scientific-P36935).

### Microscopy and image analysis

Immunostained cells were imaged on Delta Vision Core system (Applied Precision/GE Healthcare, Issaquah, WA) consisting of Olympus IX70 inverted microscope (Olympus America, Inc., Melville, NY) with 100× NA oil immersion objective and a CoolSnap HQ 12-bit camera (Photometrics, Tucson, AZ) controlled by SoftWoRX software. Filters used for imaging were DAPI (Ex360/40; Em 457/50) of the 86000 Sedat Quadruple Filter Set (Chroma Technology Corp, Bellows Falls, VT). Z-stacks of at least 10 focal planes were acquired with an exposure of 0.1–0.5 s, depending on the filter. To prepare the figures, images were deconvolved with *Softworx* and scaled manually to 8-bit using Fiji and the same range of scaling for all the images.

### RNA isolation and RT-PCR

Total RNA was extracted with TRIZOL reagent (Ambion, 15596018) and 1–2 µg of total RNA was reverse transcribed using Superscript III Reverse Transcriptase as per manufacturer instructions (Invitrogen, 18080093). PCR was performed on 1/10th volume (2 µL) of the cDNA using HotStar Taq Master Mix following manufacturer instructions (Qiagen, 203443).

#### qRT-PCR

To perform gene expression analysis using qRT-PCR, we followed the steps listed below. Initially, we calculated the mean Ct value for each sample. We then calculated ∆Ct using the formula (∆Ct = Ct Value of Target Gene - Ct Value of Endogenous Control Gene). Next, we calculated ∆∆Ct using the formula: ∆∆Ct = ∆Ct Value of Sample (Control or Treated)-Average ∆Ct of Control. Finally, we calculated the fold change (FC) using the formula FC = 2^−∆∆Ct^.

#### Cell death assay

Cells after GAPMERS treatment were detached from the plate using 200 μL trypsin and collected to the tube. Thereafter, the cells in the tubes were centrifuged for 5 min at 1,500 rpm and supernatant was discarded. The cells were washed with 2 mL PBS, and cell pellet was resuspended with 50 μL Hanks balanced salt solution. Cell suspension was mixed with 0.4% Trypan blue in a 1:1 ratio and cells were counted using a BioRad cell counter (model TC-10).

#### siRNA treatment

Double-stranded siRNAs SMART Pool (Dharmacon) were used at specified 100 nM to deplete PURPL from cell. SiRNA against Luciferase (Sigma) was used as a control at 100 nM concentrations. Lipofectamine RNAiMAX reagent (Thermo Fisher Scientific) was used for transfection as per the manufacturer’s instruction.

#### Flow activated cell sorting (FACS)

Cells were trypsinized with 0.25% Trypsin-ETDA (Himedia, TCL007) for 5 min, collected, and centrifuged at 1,500 rpm for 5 min. The cell pellet was washed with 1x PBS and fixed in ice cold methanol (100%) for overnight at 4°C. Fixed cells were pelleted and washed with 1x PBS as described above and incubated in 1x PBS containing RNase A (18 ul of 10 mg/mL RNase A + 912 ul of 1x PBS) for 30 min at room temperature. Cells were centrifuged, washed as above, and stained overnight in 1x PBS containing propidium iodide (15 µg/mL) at 4°C. The samples were analyzed by FACS (BD Biosciences FACS Aria II).

#### Statistical analysis

Error bars for all data represent SDs from the mean. All experiments were repeated at least minimum two times. P values were calculated using one-tailed type 2 Student t tests. Statistical significance is displayed as, *P < 0.05; **P < 0.01; and, ***P< 0.001.

## Results

### Treatment with reversine increased micronuclei formation along with the expression of PURPL

He et al. (2018) found that senescence happened when there were more cells in a population with abnormal chromosome numbers, specifically aneuploid and near-polyploid cells. Furthermore, chromosome missegregation causes the formation of senescent cells (Santaguida et al., 2017). We were curious to investigate whether PURPL, a gene that has been found to be increased in colorectal cancer, which is characterized by a high number of abnormal chromosomes, is associated with aneuploidy. Casella et al. (2019) demonstrated that PURPL may serve as a more resilient indicator of senescence compared to p16 mRNA or p21 mRNA. This prompted us to examine the function of PURPL in chromosomal instability (CIN) or aneuploidy.

**FIGURE 1:**
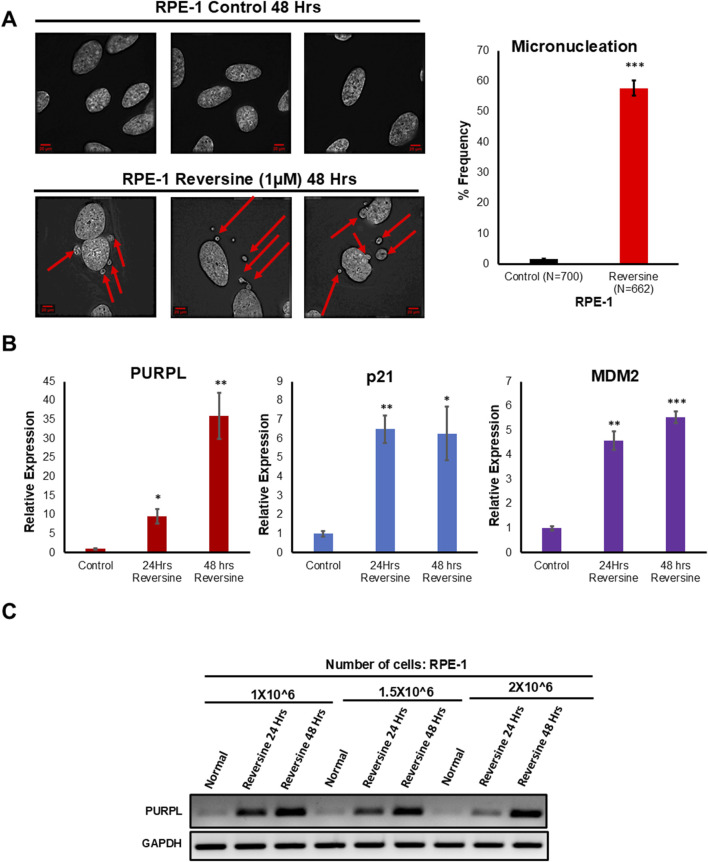
Reversine treatment leads to Chromosome Instability and increased expression of Long Noncoding RNA PURPL. **(A)** RPE-1 cells were treated with Reversine (1 µM) and incidence of micronuclei was measured after 48 h. Reversine treatment contributes to increased incidence of micronuclei in RPE-1 cells. Immunofluorescence images of RPE-1 Control upper panel and RPE-1 treated with Reversine lower panel showing presence of micronuclei in interphase cells. Red Arrows show presence of micronuclei. The graph on the right shows the quantification of micronuclei. **(B)** qRT-PCR analysis showing the expression levels of long noncoding RNA PURPL, p21 and MDM2. Increased expression of p21 and MDM2 are marker of p53 status during Chromosomal Instability. **(C)** RT-PCR showing the expression of PURPL upon Reversine treatment at different cellular densities. Error bars, SD (n = ≥2). A Student’s t-test was used. *, *P* < 0.05; **, *P* < 0.01; ***, *P* < 0.001.

Various methods have been used to induce chromosome mis-segregation in cell culture. For example, compounds that interfere with microtubule dynamics or microtubule-kinetochore attachment cause a SAC-dependent delays in mitosis and induces chromosome mis-segregation (Foley and Kapoor, 2012). When cells are exposed to inhibitors of SAC function, proper alignment of the chromosomes to the spindle is hindered resulting in generation of aneuploid progeny (Santaguida et al., 2017). We examined hTERT immortalized RPE-1 cells grown in the presence or absence of reversine at 24 and 48 h. Reversine inhibits the SAC kinase Mps1 (Zhang et al., 2016). We investigated whether Reversine treatment promotes chromosomal segregation defects in RPE-1 cells. We examined the consequences of Reversine treatment on mitosis by analyzing the incidence of micronuclei. Chromosomal Instability manifests with a higher incidence of micronuclei that often results from DNA bridges due to defective chromosome segregation. Fixed and immunostained RPE-1 ^control^ and RPE-1 ^Reversine^ cells were assayed for micronuclei due to Reversine treatment and observed that RPE-1 ^Reversine^ cells showed a higher proportion of cells with micronuclei (Figure 1A) when compared to RPE-1 ^Control^. This result indicated that Reversine treatment induces chromosomal instability. To measure the consequences of reversine on RPE-1, we did qRT-PCR to measure the gene changes associated with chromosome segregation defects. Further, we did RT -PCR and qRT-PCR to measure PURPL expression. Analysis of qRT-PCR data showed upregulation of p53 targets namely p21 and MDM2 at both 24 and 48 Hrs (Figure 1B; Supplementary Figures S1A, S1B). Once the p53 targets are validated, we measured the expression of PURPL through both RT-PCR (Figure 1C) and qRT-PCR (Figure 1B; Supplementary Figure S1C), both measurements showed increased expression of PURPL at both measured time points (Figure 1B; Supplementary Figure S1C). qRT-PCR analysis showed increased expression of PURPL along with p21 and MDM2 indicating the involvement of PURPL and p53 targets (p21 and MDM2) in chromosome segregation defects or CIN.

### Treatment of Cytochalasin-B increased the expression of PURPL

**FIGURE 2:**
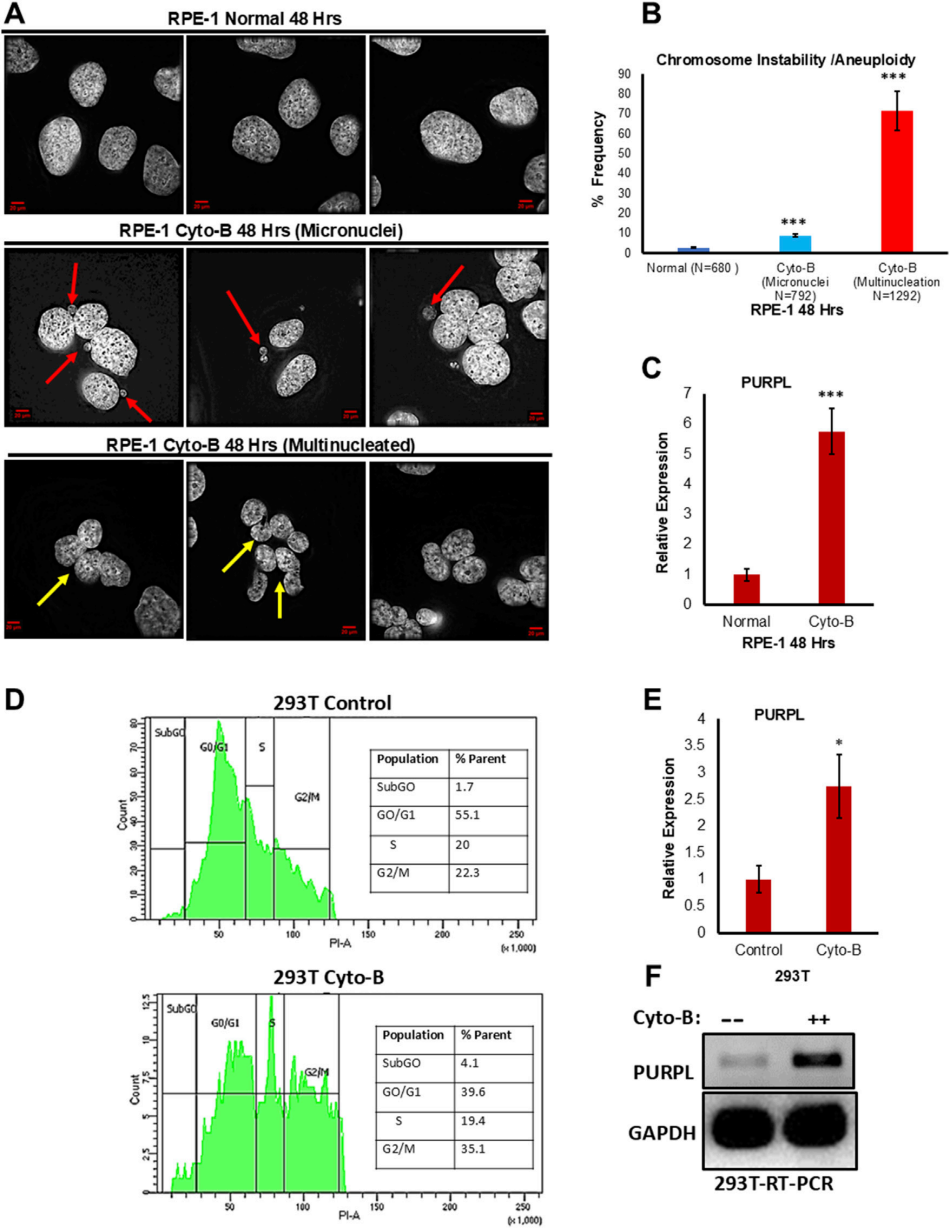
Cytochalasin-B treatment leads to Chromosome Instability/Aneuploidy and increased expression of Long Noncoding RNA PURPL. **(A)** RPE-1 cells were treated with Cytochalasin B (5 µM) and incidence of micronuclei was measured after 48 h. Cytochalasin B treatment contributes to increased incidence of micronuclei in RPE-1 cells. Immunofluorescence images of RPE-1 Normal upper panel, RPE-1 treated with Cytochalasin B middle panel showing presence of micronuclei in interphase cells and RPE-1 treated with Cytochalasin B lower panel showing presence of Multinucleation. Red Arrows show presence of micronuclei. Yellow Arrows show presence of multinucleation. **(B)** The graph on the right shows the quantification of micronuclei and multinucleation. **(C)** qRT-PCR analysis showing the expression levels of long noncoding RNA PURPL. **(D)** 293T cells were treated with Cytochalasin B (5 µM) and cell cycle was measured by flow cytometry after 72 h. Cytochalasin B treatment contributes to asynchronous cell cycle, marker for aneuploidy in 293T cells. Cell cycle profile of 293T cells control upper panel, 293T treated with Cytochalasin B lower panel showing asynchronous cell cycle. **(E)** qRT-PCR analysis showing the expression levels of long noncoding RNA PURPL. **(F)** RT-PCR showing the expression of PURPL upon Cytochalasin B treatment. Error bars, SD (n = ≥2). A Student’s t-test was used. *, *P* < 0.05; **, *P* < 0.01; ***, *P* < 0.001.

Cytochalasin B is a chemical agent which inhibits cytokinesis without preventing nuclear division (Minissi et al., 1999). Cytochalasin B was shown to induce polyploidy in several studies (Defendi and Stoker, 1973; Stanley et al., 1981). Cytokinesis failure induced in embryos through cytochalasin-B displayed segregation defects (Paim and FitzHarris, 2019). Tetraploidy perturbs metaphase chromosome alignment, kinetochore-microtubule establishment leading to CIN (Paim and FitzHarris, 2019). The other described mechanism by which tetraploidy leads to CIN in mammalian cells is that the acquisition of supernumerary centrioles/centrosomes leads to the formation of hazardous multipolar spindles resulting in segregation errors (Storchova and Kuffer, 2008). We examined hTERT immortalized RPE-1 cells grown in the presence or absence of Cytochalasin-B at 24 and 48 h. We investigated whether Cytochalasin B treatment promotes chromosomal instability defects in RPE-1 cells. We examined the consequences of Cytochalasin B treatment by analyzing the incidence of micronuclei. We scored for cells with the incidence of micronuclei. Fixed and immunostained RPE-1 ^control^ and RPE-1 ^Cytochalasin−B^ cells were assayed for incidence of micronuclei status due to cytochalasin-B treatment and observed that RPE-1 ^Cytochalasin B^ cells showed a higher proportion of cells with micronuclei (Figures 2A, B; Supplementary Figures S2A, S2B), further supporting the conclusion that Cytochalasin-B treatment contribute to CIN. Cell multinucleation is closely associated with chromosomal instability (Pantelias et al., 2019). Polyploidy cells were shown to have defective chromosome segregation resulting in aneuploidy (Potapova et al., 2013). Furthermore, polyploidy is also associated with high levels of chromosome instability (Mayer and Aguilera, 1990). Fixed and immunostained RPE-1 ^control^ and RPE-1 ^Cytochalasin−B^ cells were assayed for incidence of multinucleation due to cytochalasin-B treatment and observed that RPE-1 ^Cytochalasin B^ cells showed a higher proportion of multinucleated cells (Figures 2A, B; Supplementary Figures S2A, S2B), further supporting the conclusion that Cytochalasin-B treatment contributes to CIN. Treatment with Cytochalasin-B led to micronuclei formation and polyploidy generation in a time-dependent manner (Figure 2A; Supplementary Figure S2A). To measure the consequences of Cytochalasin-B on PURPL expression, we did qRT-PCR to measure PURPL, p21, and MDM2 expression. The qRT-PCR measurements showed increased expression of PURPL at both measured time points (Figure 2C; Supplementary Figure S2C). In addition, qRT-PCR showed increased expression of p21 and MDM2 at 48 h, but at 24 h, only MDM2 showed increased expression, but p21 expression did not change (Supplementary Figure S2D). These results indicated the involvement of PURPL and p53 targets (p21 and MDM2) in chromosome segregation defects or CIN.

In order to verify whether the increased expression of PURPL upon cytochalasin-B treatment is cell line-specific, we treated 293T cells with cytochalasin-B and measured PURPL expression. Chromosomal instability and aneuploidy are associated with cell cycle defects, resulting in an asynchronous cell cycle. We measured the cell cycle profile of 293T cells treated with or without cytochalasin-B through flow cytometry. 293T cells treated with cytochalasin-B resulted in an asynchronous cell cycle (Figure 2D lower panel) as compared to control cells (Figure 2D upper panel), further supporting the conclusion that cytochalasin-B treatment contributes to CIN, or aneuploidy. To measure the consequences of Cytochalasin-B on PURPL expression in 293T cells, we did qRT-PCR and RT-PCR to measure PURPL expression. The qRT-PCR measurements showed increased expression of PURPL at 72 h (Figure 2E). This result was further validated through increased expression of PURPL through RT-PCR (Figure 2F). These results indicated the involvement of PURPL in CIN or aneuploidy, and this phenomenon is not cell line-specific.

### Treatment of Aurora kinase inhibitor (ZM447439) increased the expression of PURPL

ZM447439 (“ZM”), the first Aurora family kinase inhibitor to be developed and characterized was previously found to interfere with the mitotic spindle integrity checkpoint and chromosome segregation (Gadea and Ruderman, 2005). When ZM was added to mammalian somatic tissue culture cells, the cells mitotic spindle was disorganized, chromosomes did not align properly, and cytokinesis was blocked (Gadea and Ruderman, 2005).

**FIGURE 3:**
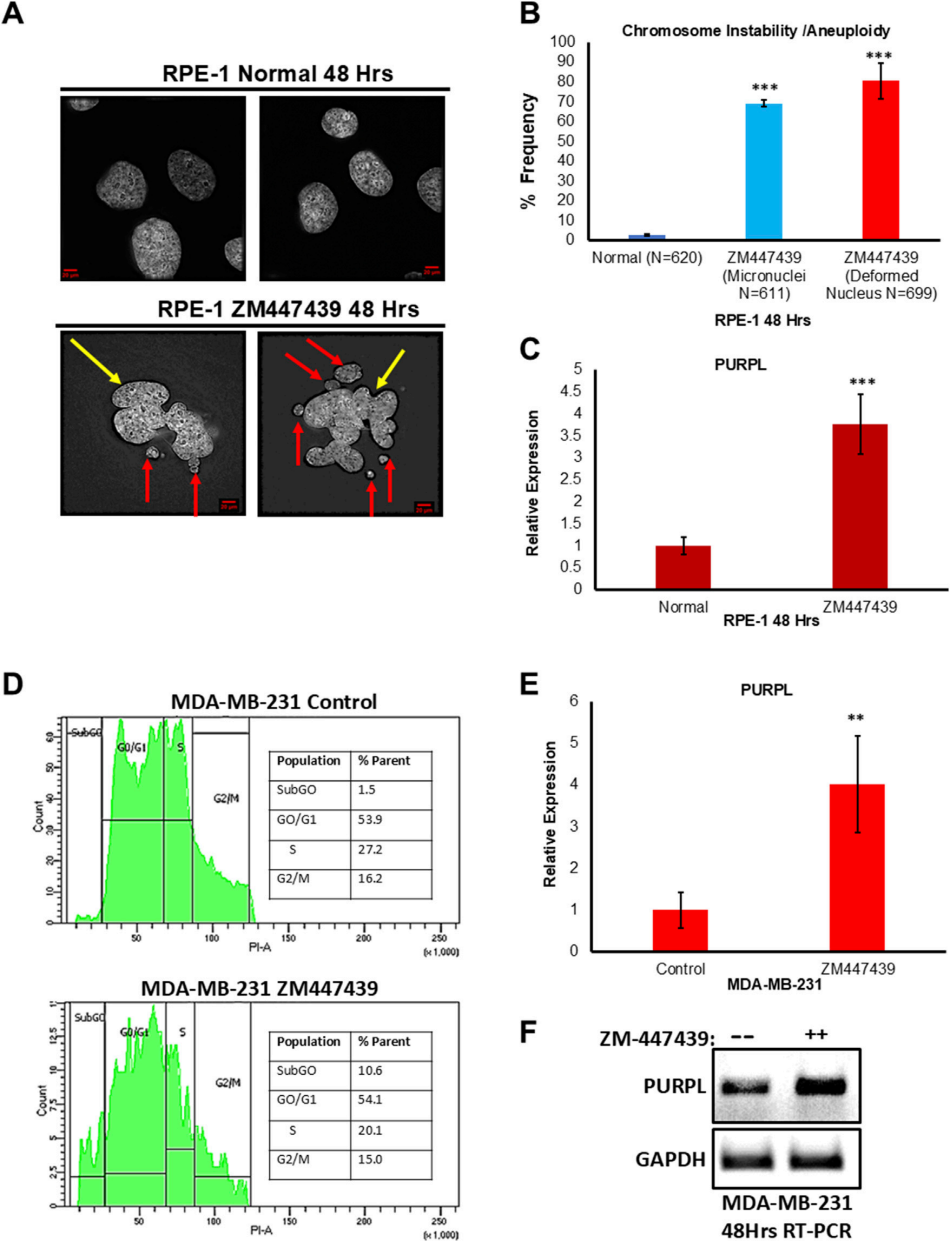
Aurora Kinase Inhibitor (ZM447439) treatment leads to Chromosome Instability/Aneuploidy and increased expression of Long Noncoding RNA PURPL. **(A)** RPE-1 cells were treated with ZM447439 (10 µM) and incidence of micronuclei and deformed nucleus was measured after 48 h. ZM447439 treatment contributes to increased incidence of micronuclei and deformed nucleus in RPE-1 cells. Immunofluorescence images of RPE-1 Normal upper panel, RPE-1 treated with ZM447439 lower panel showing presence of micronuclei and deformed nucleus. Red Arrows show presence of micronuclei. Yellow Arrows show presence of deformed nucleus. **(B)** The graph on the right shows the quantification of micronuclei and deformed nucleus. **(C)** qRT-PCR showing the expression levels of long noncoding RNA PURPL. **(D)** MDA-MB-231 cells were treated with ZM447439 (10 µM) and cell cycle was measured by flow cytometry after 72 h. ZM447439 treatment contributes to asynchronous cell cycle, marker for aneuploidy in 293T cells. Cell cycle profile of MDA-MB-231 control upper panel, MDA-MB-231 treated with ZM447439 lower panel showing asynchronous cell cycle. **(E)** qRT-PCR analysis showing the expression levels of long noncoding RNA PURPL. **(F)** RT-PCR showing the expression of PURPL upon Cytochalasin B treatment. Error bars, SD (n = ≥2). A Student’s t-test was used. *, *P* < 0.05; **, *P* < 0.01; ***, *P* < 0.001.

We examined the consequences of ZM447439 treatment by analyzing the incidence of micronuclei. We scored for cells with the incidence of micronuclei. Fixed and immunostained, RPE-1 ^control^, and RPE-1 ^ZM447439^ cells were assayed for incidence of micronuclei status due to ZM447439 treatment and observed that RPE-1 ^ZM447439^ cells showed a higher proportion of cells with micronuclei (Figure 3A; Supplementary Figure S3A), further supporting the conclusion that ZM447439 treatment contributes to CIN. Abnormal nuclear shape and chromosomal instability are closely related (Gisselsson et al., 2001). A linear and positive correlation was observed between the abnormal nuclear shape on one hand and cells with chromosomal instability feature like unstable chromosomes and anaphase bridges on the other hand (Gisselsson et al., 2001). Fixed and immunostained RPE-1 ^control^, and RPE-1 ^ZM447439^ cells were assayed for incidence of abnormal nuclear shape due to ZM447439 treatment and observed that RPE-1 ^ZM447439^ cells showed a higher proportion of abnormal nuclear shape (Figures 3A, B; Supplementary Figures S3A, S3B), further supporting the conclusion that ZM447439 treatment contributes to CIN. Treatment with ZM447439 showed chromosome instability features in a time-dependent manner (Figure 3A; Supplementary Figure S3A). To measure the consequences of ZM447439 on RPE-1, we did qRT-PCR to measure PURPL, p21 and MDM2 expression, indeed qRT-PCR showed increased expression of p21 and MDM2 at both 24 and 48 h (Supplementary Figures S3B, S3C). Once the p53 targets are validated, we measured the expression of PURPL through qRT-PCR at both measured time points. qRT-PCR analysis showed increased expression of PURPL (Figure 3C; Supplementary Figure S3B) indicating the involvement of PURPL and p53 targets (p21 and MDM2) in CIN or aneuploidy.

In order to verify whether the increased expression of PURPL upon ZM447439 treatment is cell line-specific, we treated MDA-MB-231 cells with ZM447439 and measured PURPL expression. Chromosomal instability and aneuploidy are associated with cell cycle defects, resulting in an asynchronous cell cycle. We measured the cell cycle profile of MDA-MB-231 cells treated with or without ZM447439 through flow cytometry. MDA-MB-231 cells treated with cytochalasin-B resulted in an asynchronous cell cycle (Figure 3D lower panel) as compared to control cells (Figure 3D upper panel), further supporting the conclusion that ZM447439 treatment contributes to CIN, or aneuploidy. To measure the consequences of ZM447439 on PURPL expression in MDA-MB-231 cells, we did qRT-PCR and RT-PCR to measure PURPL expression. The qRT-PCR measurements showed increased expression of PURPL at 72 h (Figure 3E). This result was further validated through increased expression of PURPL through RT-PCR (Figure 3F). These results indicated the involvement of PURPL in chromosome CIN or aneuploidy, and this phenomenon is not cell line-specific.

### PURPL expression is dependent on p53 expression

**FIGURE 4:**
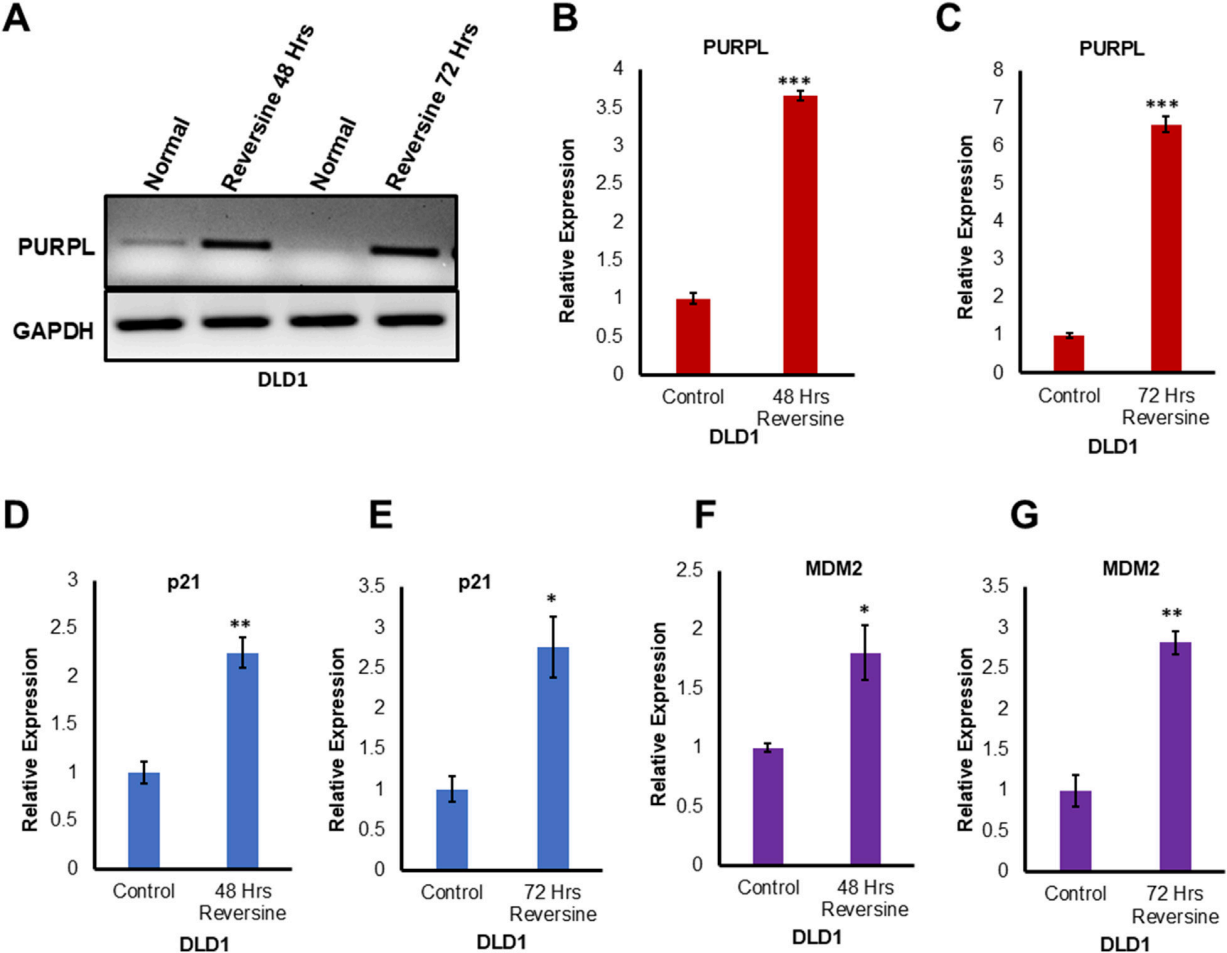
Reversine known inducer of Chromosome Instability treatment leads to increased expression of Long Noncoding RNA PURPL in DLD1 cells. **(A)** DLD1 cells were treated with Reversine (1 µM) and expression of PURPL was measured after 48 and 72 h. RT-PCR showing the expression of PURPL upon Reversine treatment. **(B–G)** qRT-PCR showing the expression levels of long noncoding RNA PURPL, p21 and MDM2 after 48 and 72 h of reversine treatment. Increased expression of p21 and MDM2 are marker of p53 status during Chromosomal Instability. Error bars, SD (n = ≥2). A Student’s t-test was used. *, *P* < 0.05; **, *P* < 0.01; ***, *P* < 0.001.

PURPL was shown to be upregulated upon DOXO treatment in a p53-dependent manner (Li et al., 2017). p53-dependent upregulation of PURPL was further strengthened by qRT-PCR upon genetic loss of p53 or upon p53 knockdown in HCT116 in the presence or absence of DOXO (Li et al., 2017). PURPL, an abundant lncRNA upregulated after DOXO treatment was abolished upon genetic loss of 53 or upon p53 knockdown (Li et al., 2017). In order to look at the role of p53 in the regulation of PURPL expression due to CIN, we looked at the DLD1 cells. DLD1 is a pseudodiploid human cell line with p53 mutation (Galofré et al., 2020). The p53 protein produced in DLD1 cells has a point mutation at position 241 (C -> T a mutation resulting in Ser -> Phe). We examined DLD1 cells grown in the presence or absence of reversine at 48 and 72 h. We did RT-PCR and qRT-PCR to measure PURPL expression, indeed RT-PCR (Figure 4A) and qRT-PCR (Figures 4B, C) showed increased expression of PURPL at both 48 and 72 h (Figures 4A–C). Once the PURPL exression was validated, we measured the expression of p53 targets through qRT-PCR. qRT-PCR (Figures 4D–G) measurement showed increased expression of p21 and MDM2 at both measured time points. The magnitude of PURPL, p21, and MDM2 expression was less than that observed in the case of RPE-1 cells treated with Reversine (Figures 4, 1B; Supplementary Figure S1). The decreased expression of PURPL along with p21 and MDM2 in DLD1 cells indicate the involvement of p53 in PURPL expression in CIN or aneuploidy.

**FIGURE 5:**
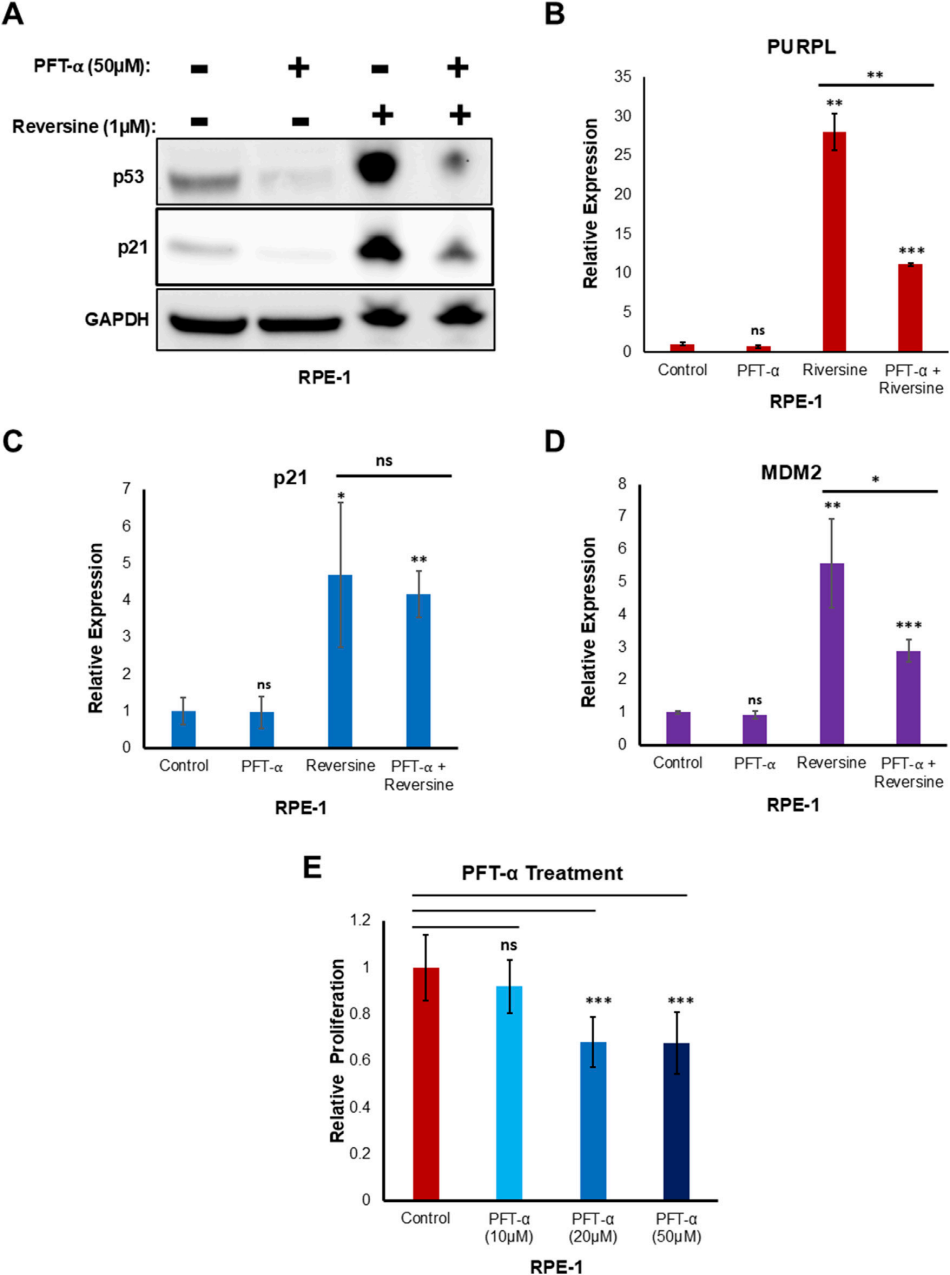
Reversine treatment in presence of PFT- α resulted in reduced expression of Long Noncoding RNA PURPL. Pifithrin-α (PFT- α) is an inhibitor of p53, inhibiting p53-dependent transactivation of p53-responsive genes. **(A)** Western blot showing the levels of indicated proteins. GAPDH was used as loading control. **(B)** qRT-PCR showing the expression levels of long noncoding RNA PURPL. **(C–D)** qRT-PCR showing the expression levels of p21, and MDM2. **(E)** The graph displays the relative proliferation of cells treated with and without PFT-α. We normalized the proliferation of control cells to 1, and plotted the proliferation of cells treated with different doses of PFT-α in comparison to the control cells. Error bars, SD (n = ≥2). A Student’s t-test was used. *, *P* < 0.05; **, *P* < 0.01; ***, *P* < 0.001.

To validate the p53-dependent upregulation of PURPL by reversine through qRT-PCR, RPE-1 cells were treated with reversine, in the presence or absence of Pifithrin-α. Pifithrin-α is a reversible inhibitor of p53 (Zhu et al., 2020). Reversine treatment resulted in increased expression of PURPL in absence of a p53 inhibitor (Figures 5A, B; Supplementary Figures S4A, S4B; Supplementary Figures S4C, S4D). Furthermore, in presence of Pifithrin-α (50 uM), reversine treatment resulted in a weakened response of increased expression of PURPL (Figures 5A, B; Supplementary Figures S4C, S4D).

The increased expression of PURPL did not decrease much in the presence of 10 μM and 20 µM of Pifithrin-α (Supplementary Figures S4A, S4B). Furthermore, the increased transcriptional activation of p21 upon reversine treatment did not show any change in the expression with and without Pifithrin-α (50 uM) (Figure 5C). In addition, qRT-PCR analysis showed weakened response of increased expression of MDM2 in presence of reversine along with Pifithrin-α (50 uM) (Figure 5D). These results indicated that the strength or magnitude of increased expression of PURPL upon reversine treatment is dependent upon p53 and its target MDM2.

To see if the lower level of PURPL in cells treated with reversine after p53 knockdown was because of PFT-α′s effect on proliferation, we looked at how PFT-α affected proliferation. 10 μM of PFT-α did not show any significant effect on proliferation (Figure 5E). 20 µM and 50 µM of PFT-α showed some effect on proliferation (Figure 5E), but this could not be corroborated with the effect on PURPL expression upon reversine treatment. At both 20 µM and 50 µM of PFT-α treatment, there was a similar reduced effect on proliferation (Figure 5E), but the effect on PURPL expression was greater for 50 µM of PFT-α as compared to 20 µM (Supplementary Figures S4A, S4C). The results (Figure 5; Supplementary Figure S4) did not show a direct link between the lower levels of PURPL after reversine treatment for cells that had been treated with 50 µM of PFT-α and the lower cell growth that 50 µM of PFT-α caused.

### PURPL loss of function results in micronuclei formation, deformed nuclear shape, and increased sensitivity towards CIN or aneuploidy inducers

**FIGURE 6:**
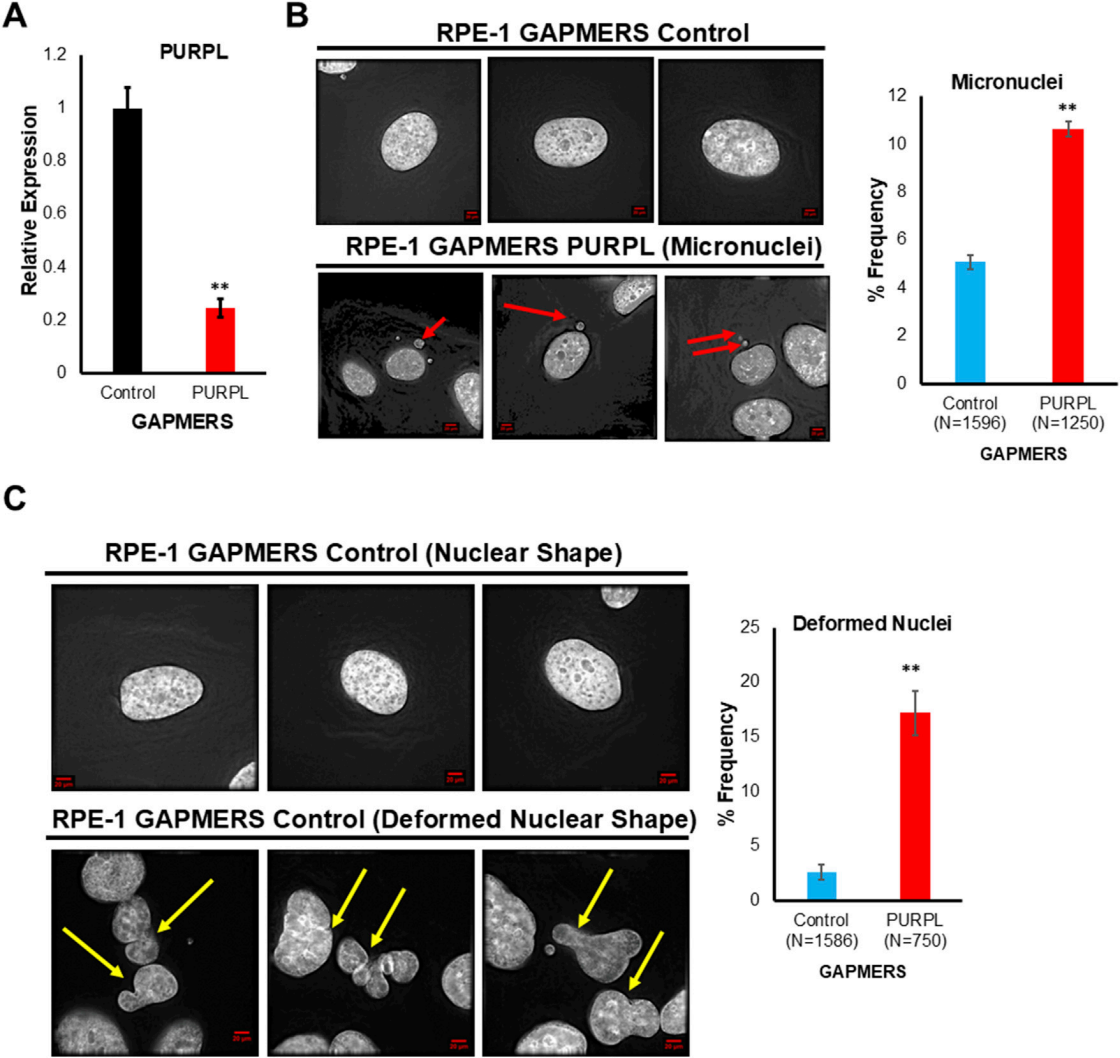
Long Noncoding RNA, PURPL knockdown contributes to Chromosomal Instability. **(A)** Knockdown of PURPL in RPE-1 Cells using GAPMERS. qRT-PCR showing the expression of PURPL. **(B)** PURPL knockdown contributes to increased incidence of micronuclei in RPE-1 cells. Immunofluorescence images of RPE-1 control GAPMERS upper panel **(B)** and RPE-1 PURPL GAPMERS showing presence of micronuclei in interphase cells. Red Arrows show presence of micronuclei. The graph on the right shows the quantification of micronuclei. **(C)** PURPL knockdown contributes to increased incidence of nuclear defects in RPE-1cells. Immunofluorescence images of RPE-1 control GAPMERS (upper panel) and RPE-1 PURPL GAPMERS (lower Panel) showing nuclear morphology. Yellow arrows show deformed nucleus. The graph on the right shows the quantification of deformed nucleus. Error bars, SD (n = ≥2). A Student’s t-test was used. *, *P* < 0.05; **, *P* < 0.01; ***, *P* < 0.001.

To elucidate potential functions of PURPL, we knockdown PURPL with GAPMERS and looked for its role on chromosomal instability in RPE-1 cells. The efficiency of the PURPL knockdown was checked through qRT-PCR. Use of PURPL GAPMERS resulted in almost 80% knockdown of PURPL as compared to control (Figure 6A). We examined the consequences of PURPL knockdown by analyzing the incidence of micronuclei and deformed nuclei. We scored for cells with defective segregation as depicted by the presence of deformed nucleus and incidence of micronuclei. Fixed and immunostained RPE-1 GAPMERS ^Control^, and RPE-1 ^GAPMERS PURPL^ cells were assayed for micronuclei due to PURPL knockdown and observed that RPE-1 ^GAPMERS PURPL^ cells displayed a significant increase in the proportion of cells with micronuclei as compared to RPE-1 GAPMERS ^Control^ (Figure 6B). Hence, we concluded that PURPL knockdown correlates with defects in chromosome segregation as shown by an increased incidence of micronuclei. In addition to the incidence of micronuclei, CIN also manifests abnormal nuclear shape (Gisselsson et al., 2001). A positive correlation was observed between the abnormal nuclear shape and chromosomal instability features like unstable chromosomes and anaphase bridges (Gisselsson et al., 2001). Fixed and immunostained RPE-1 GAPMERS ^Control^ and RPE-1 ^GAPMERS PURPL^ cells were assayed for nuclear shape due to downregulation of PURPL and observed that RPE-1 ^GAPMERS PURPL^ cells displayed a significant increase in the proportion of cells with a deformed nucleus (Figure 6C). Cells with PURPL knockdown, developed a higher proportion of misshapen, multilobed nuclei, and contained micronuclei. These morphological abnormalities were phenocopies of siRNA-mediated reduction in centromeric proteins itself that drives chromosome mis-segregation events underlying the interphase nuclear defects. The nuclei of normal cells are normally ellipsoid shapes with smooth outlines (Gisselsson et al., 2001). Deformed nuclei are characteristics of many cancer cells where they are easily identifiable by increased nuclear size, irregular nuclear contours, and disturbed chromatin distribution, making nuclear morphology one of the oldest and most commonly used cancer markers (Gisselsson et al., 2001; De las Heras et al., 2013; Denais and Lammerding, 2014). The irregular nuclear outline in cancer cells is mainly the result of grooving, convolutions, and invaginations of the nuclear envelope (Van Bortle and Corces, 2012; De las Heras et al., 2013; Denais and Lammerding, 2014). These data indicate that PURPL loss results in perturbing nuclear architecture ultimately leading to chromosomal instability with the potential to play an important role in cancer. Furthermore, these results indicated that proper levels of PURPL expression are critical for organized nuclear architecture. These studies indicate that expression levels of PURPL must be precisely regulated for maintaining genomic instability.

**FIGURE 7:**
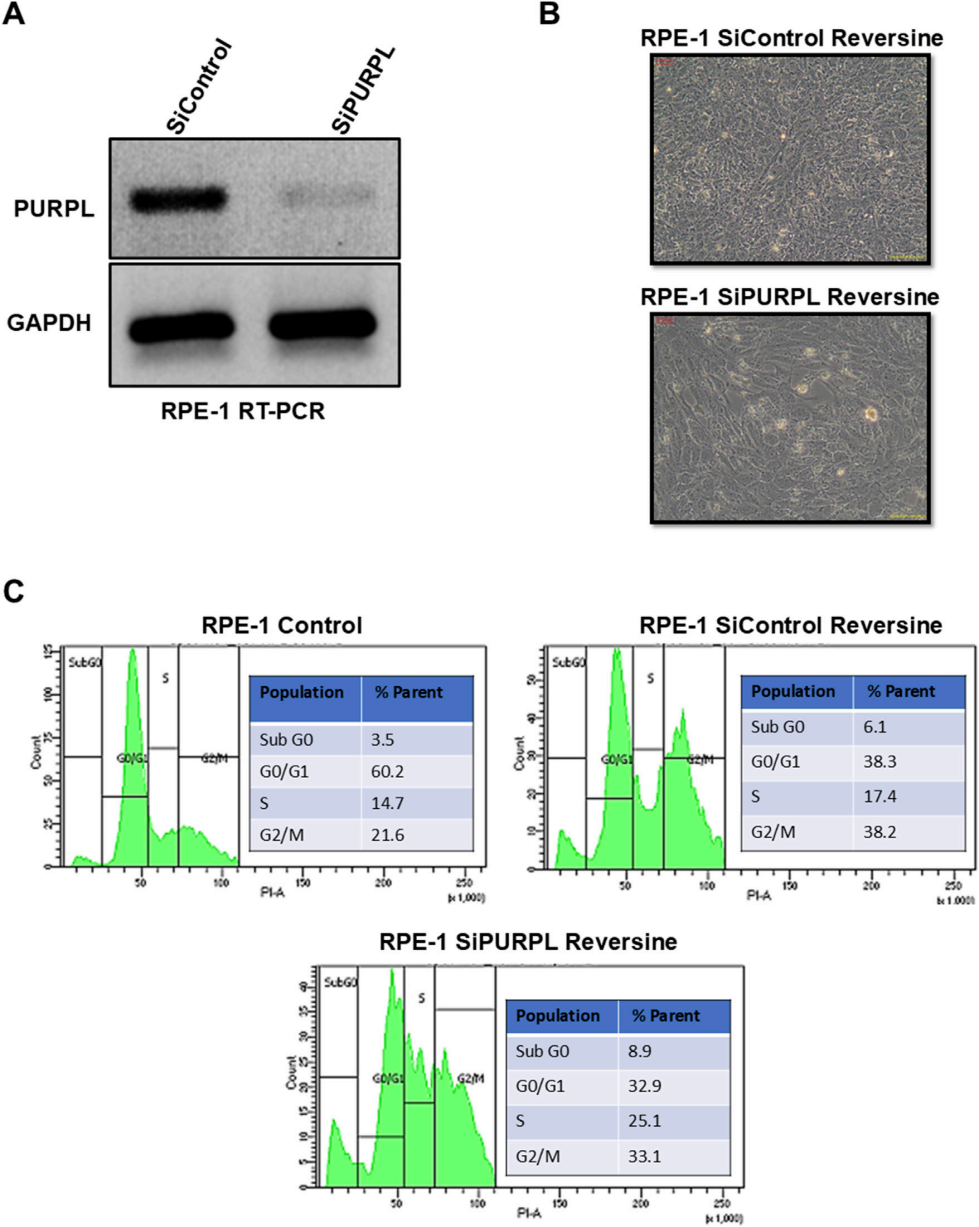
PURPL knockdown increases RPE-1’s sensitivity to aneuploidy after reversine treatment. **(A)** RT-PCR showing the knockdown of lncRNA PURPL in RPE-1 cells. **(B)** Microscopic images showing the morphology of cells treated with reversine (with SiRNAs against luciferase as a control or lncRNA PURPL). **(C)** The FACS profile illustrates the various phases of the cell cycle, accompanied by a quantification of the percentage of cells in each phase for the cells outlined in **(B)**.

**FIGURE 8:**
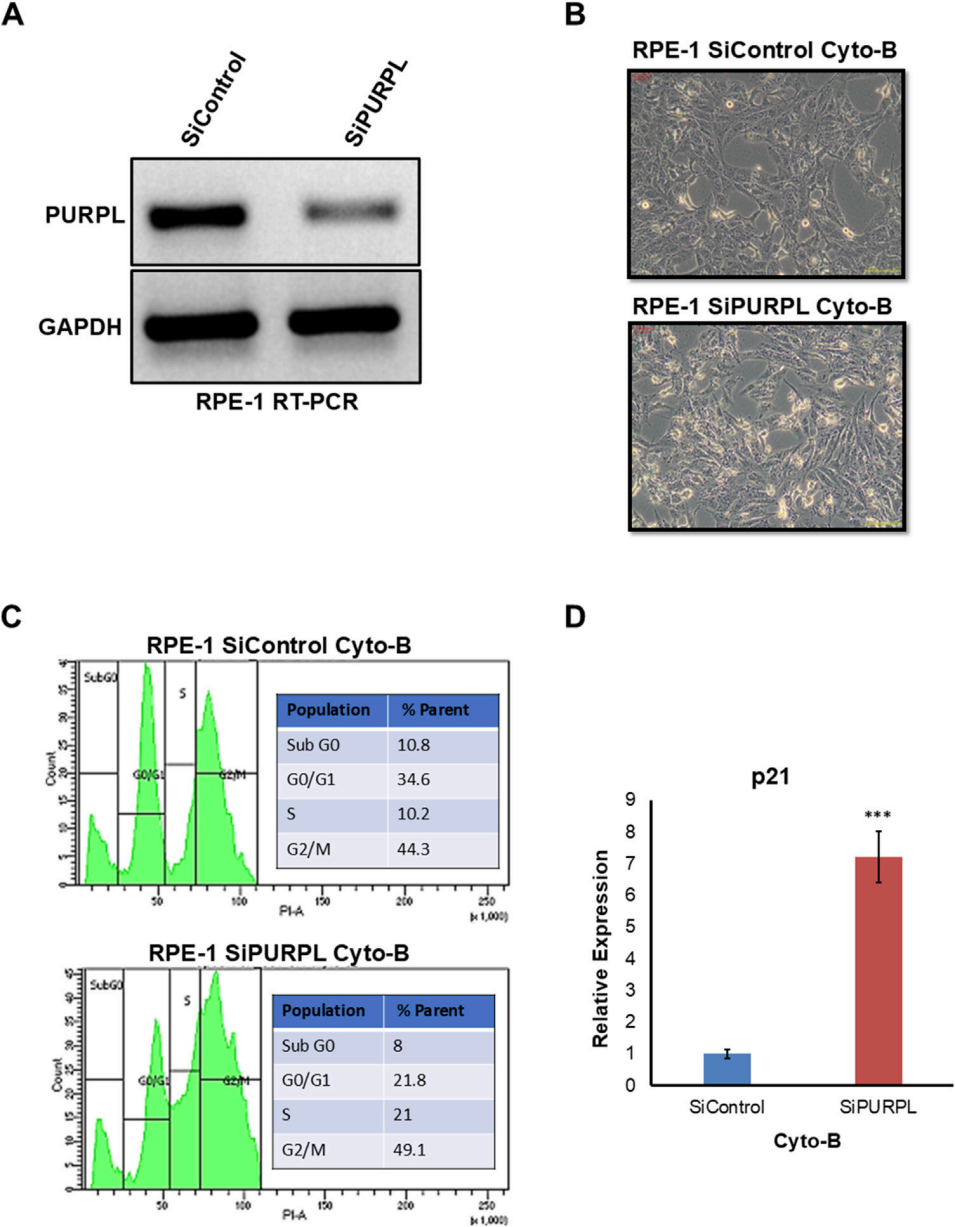
PURPL knockdown increases RPE-1’s sensitivity to aneuploidy following cytochalasin-B treatment. **(A)** RT-PCR showing the knockdown of lncRNA PURPL in RPE-1 cells. **(B)** Microscopic images showing the morphology of cells that were treated with SiRNAs against luciferase as a control or against lncRNA PURPL for 48 h and then treated with Cytochalasin-B for 24 h. **(C)** The FACS profile illustrates the various stages of the cell cycle, accompanied by a quantification of the percentage of cells in each phase for the cells outlined in **(B)**. **(D)** The qRT-PCR data reveals the expression level of p21 in the cells referenced in **(B)**. We used actin expression to normalize the data.

**FIGURE 9:**
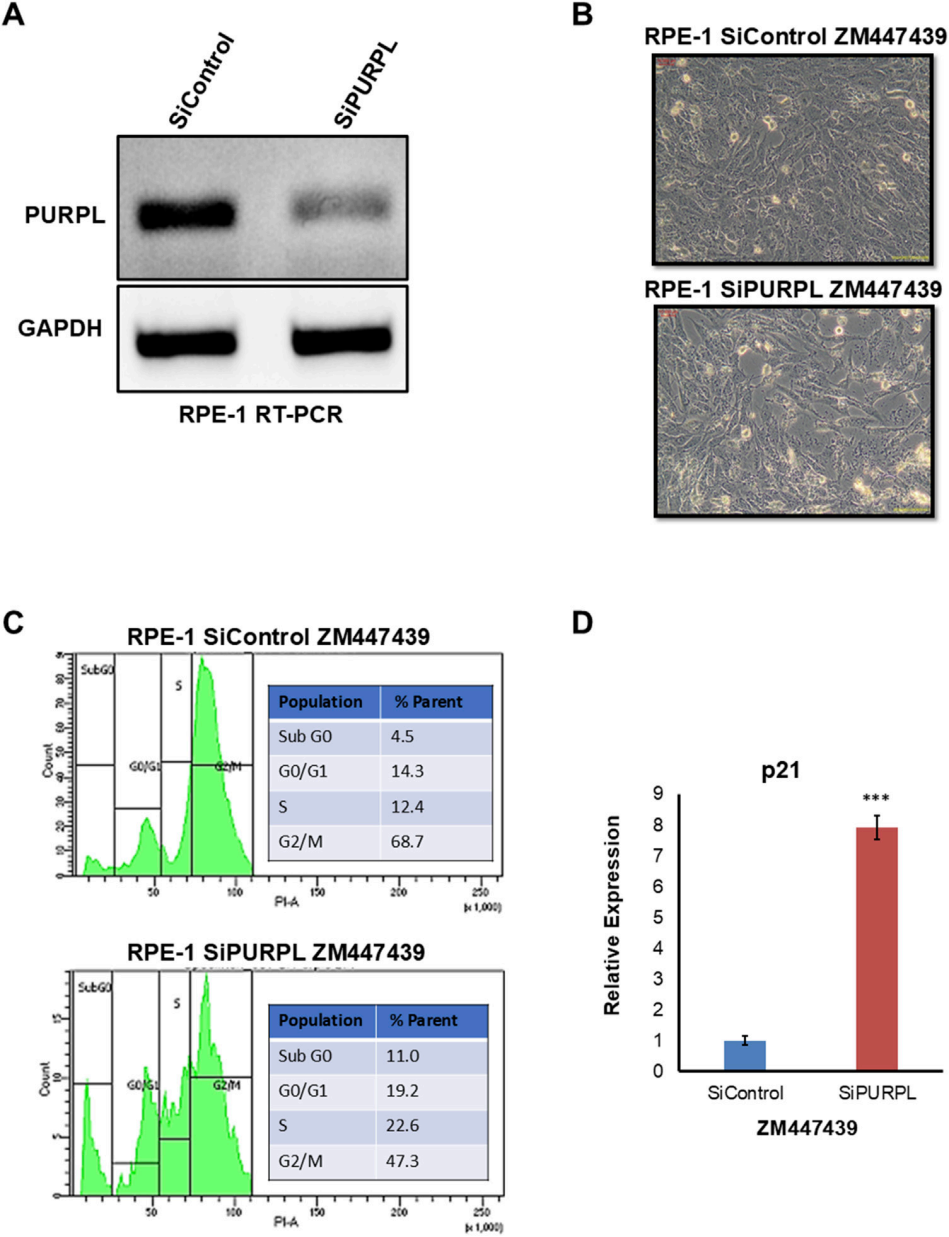
PURPL knockdown increases RPE-1’s sensitivity to aneuploidy following ZM447439 treatment. **(A)** RT-PCR showing the knockdown of lncRNA PURPL in RPE-1 cells. **(B)** Microscopic images showing the morphology of cells treated with SiRNAs against luciferase as a control or against lncRNA PURPL for 48 h, followed by treatment with ZM447439 for 24 h. **(C)** The FACS profile illustrates the various stages of the cell cycle, accompanied by a quantification of the percentage of cells in each phase, for the cells outlined in **(B)**. **(D)** qRT-PCR data showing the expression level of p21 for cells described in **(B)**. The expression of actin was used for normalization.

When we knocked down PURPL, we observed a change in the nucleus’ shape and an increase in micronuclei, prompting us to investigate its role in CIN/aneuploidy. We transiently knocked down the expression of PURPL in RPE-1 cells (Figures 7–9) and then compared how well they did when treated with CIN or aneuploidy inducers like reversine, cytochalasin-B, or ZM447439 (Figures 7–9).

To cause aneuploidy, cells with or without PURPL knockdown were given 1 µM Reversine, 2.5 µM Cytochalasin-B, and 10 µM ZM447439 for 24 h. PURPL knockdown cells showed morphological changes as compared to control cells. Cells with PURPL knockdown were more elongated and stressed as compared to control cells upon reversine treatment, showing that reversine is inducing more changes in PURPL knockdown cells (Figures 7A, B). In addition, the cells are more stressed, with clear morphological changes following cytochalasin-B treatment (Figures 8A, B). Treatment with ZM447439 also induces more morphological changes in PURPL knockdown cells (Figures 9A, B). PURPL knockdown cells appear more elongated and stressed (Figures 9A, B).

Studies have shown that cells with chromosome instability or aneuploidy have more S-phase (Look et al., 1982; Gemble et al., 2022) or sub-G0 phase cells (Santaguida et al., 2017) in the cell cycle compared to control cells. FACS is a quantitative way to measure cells in different phases of the cell cycle. The flow cytometry analysis of PURPL knockdown cells showed that they had more Sub G0 (8.9% vs. 6.1%) and S-Phase (25.1% vs. 17.4%) cells than control cells upon reversine treatment (Figure 7C). Cytochalasin-B treatment increased the proportion of S-phase cells to 21% from 10.2% in comparison to control cells (Figure 8C). Once ZM447439 was added to PURPL knockdown cells, there were more cells in Sub G0 (11% vs. 4.5%) and S-Phase (22.6% vs. 12.4%) (Figure 9C). These results clearly indicate that PURPL knockdown cells are more vulnerable to aneuploidy as compared to control cells.

We checked the level of p21 due to its association with chromosomal instability or aneuploidy (Kumari et al., 2014). Once we treated PURPL knockdown cells with cytochalasin-B (Figure 8D) and ZM447439 (Figure 9D), we saw that they expressed more p21 than control cells. These results clearly indicate that PURPL knockdown cells are more susceptible to chromosomal instability, or aneuploidy, as compared to control cells.

### PURPL regulates chromosomal instability or aneuploidy, likely through MDM2

In order to find the molecular mechanism by which PURPL can regulate nuclear morphology or chromosomal stability, we focused on MDM2 for the following reasons: The MDM2 gene was subsequently found to be amplified in cancer (Oliner et al., 2016). MDM2 is a negative regulator of the tumor suppressor p53 (Momand et al., 2000). Elevated MDM2 expression was shown to induce chromosomal instability in aging mice (Lushnikova et al., 2011). In addition, it was also shown to induce chromosomal instability when overexpressed in B cells (Wang et al., 2007). The capability of overexpressed MDM2 to affect genomic instability can also happen independently of p53 (Bohlman and Manfredi, 2014). PURPL was shown to be regulated by p53 and, at the same time, regulate the expression of p53 (Li et al., 2017). Since both PURPL and MDM2 are shown to be regulated by p53 and, in turn, they regulate the expression of p53, we looked at the regulation of MDM2 by PURPL. We looked at the expression of MDM2 upon transient knockdown of PURPL using GAPMERS in RPE-1 cells (Supplementary Figure S5A). Knockdown of PURPL using GAPMERS resulted in increased cell death in RPE-1 cells (Supplementary Figure S5B). qRT-PCR analysis showed knockdown of PURPL resulted in increased expression of MDM2 (Supplementary Figure S5C). This result suggested that PURPL regulates the expression of MDM2. Further, we used a bioinformatics tool called catRAPID to predict MDM2 and PURPL interactions (Agostini et al., 2013). catRAPID predicted an interaction propensity of 0.94 between PURPL and MDM2, suggesting an average probability of interaction between PURPL and MDM2 (Supplementary Figure S5D). The interaction profile of the nucleotide position of long noncoding RNA PURPL with MDM2 showed that the nucleotide position from 400 to 1,000 nucleotides showed more probability of interaction with MDM2 as compared to the first 400 nucleotides (Supplementary Figure S5E). In addition, an interaction matrix involving long noncoding RNA PURPL nucleotide positions and individual protein residue fragments of MDM2 indicated that MDM2 fragments from 100 to 450 amino acid residues have a greater chance of interacting with 400–1,000 nucleotides of PURPL (Supplementary Figure S5F). These bioinformatics analyses indicate that PURPL may interact with MDM2 directly, although more extensive biochemical characterization is required to confirm this. Through probable interactions with MDM2, PURPL may regulate MDM2 expression. The knockdown of PURPL led to an increase in MDM2 expression, potentially explaining the observed increase in CIN or aneuploidy and subsequent cell death.

## Discussion

Here, we report the initial functional characterization of PURPL in the context of chromosomal instability or aneuploidy. Our studies of this lncRNA have yielded several important and unexpected findings. We report the expression profile of lncRNA PURPL in three experimental models of chromosomal instability or aneuploidy. We identified PURPL overexpression shared among all comparison pairs of chromosomal instability or aneuploidy inducers. Using microscopy, FACS to score CIN or aneuploidy and qRT-PCR to measure PURPL expression showed that the CIN or aneuploidy phenotypes and PURPL expression depend more on how they were induced. For example, reversine-induced CIN or aneuploidy and PURPL expression are higher as compared to CIN or aneuploidy triggered by other stimuli. The dissimilarity between the extent of PURPL expression and CIN/aneuploidy features upon reversine, cytochalasin B, and ZM447439 could be due to differences in the dynamic progression of RPE-1 towards chromosomal instability or aneuploidy and the extent of CIN or aneuploidy induced by different agents.

In response to chromosomal instability or aneuploidy, our studies have identified the lncRNA PURPL as a component of the chromosome missegregation response, upregulating it along with p53 activity. Activation of the p53 pathway upon reversine treatment is in agreement with previous studies where it was reported that chromosome missegregation causes p53 activation and a p53-dependent cell cycle arrest (Kuffer et al., 2013; Santaguida et al., 2017). This p53 activation or PURPL overexpression could be caused by aneuploidy itself or by events that happen when chromosomes do not get sorted correctly. This result also explains the observed upregulation of PURPL in colorectal cancers, which are characterized by extensive aneuploidy, or CIN. Interestingly, it was recently reported that PURPL is associated with senescence, suggesting broader roles for PURPL in cellular stress responses (Casella et al., 2019). Future research should look into how PURPL affects the functions of CIN or genomic instability and other stress response pathways. It should also look into the bigger roles of PURPL in health and illness.

Our results of reduced PURPL induction in DLD1 (mutated p53) cells treated with reversine indicated that the expression of PURPL is dependent on p53. Further our result of PURPL expression in RPE-1 cells treated with Reversine in presence of p53 inhibitor indicated that p53 regulates the strength of induction of PURPL though not solely responsible. The result of p53 regulating the magnitude of PURPL expression is in agreement with the previous study of PURPL expression regulation by p53 in the presence of doxorubicin treatment (Li et al., 2017). In the study mentioned, in the absence of p53 (p53KO), there is almost no induction of PURPL in the presence of doxorubicin (Li et al., 2017). Furthermore, the present study clearly indicated that the response of PURPL to CIN-inducing reagents like reversine is not solely dependent on p53. The other possible reason for PURPL expression in RPE-1 cells treated with reversine in the presence of a p53 inhibitor could be the phenomenon of senescence-associated chromosomal instability. Eight different models of senescence showed upregulation of PURPL (Casella et al., 2019).

Here, we present the functional characterization of a low-expression PURPL lncRNA in mammalian cells and tissues. Our studies of this lncRNA have yielded important and unexpected findings. Inactivation of PURPL is sufficient to produce a CIN phenotype in previously karyotypically stable cell lines, revealing an essential role for a lncRNA in maintaining chromosomal stability in mammalian cells. We also propose that PURPL might preserve genomic stability by controlling the expression of MDM2, which is a negative regulator of tumor suppressor p53-binding proteins. This is likely to occur through PURPL effects on MDM2. The direct involvement of MDM2 in CIN has been previously demonstrated by showing that overexpression of MDM2 could elicit genomic instability (Wang et al., 2007; Lushnikova et al., 2011). Thus, it is plausible that MDM2 acts as a mediator of the PURPL-induced CIN phenotype.

However, it should be noted that although CIN or aneuploidy can be the underlying mechanism for PURPL-promoted tumor growth, the mechanistic connections between these phenotypes are not established in this study. In this study, we showed that proper levels of PURPL expression are critical for maintaining genome organization or chromosomal stability. Knockdown of PURPL resulted in a defective CIN or aneuploid phenotype. These studies indicated that expression levels of PURPL must be precisely regulated for normal nuclear organization or chromosomal stability in cells. We think in this case it is because the function of PURPL has to be in exact concentrations in order to function correctly, so reducing it would have a negative effect. The observed results are also in agreement with knockdown studies of several centromeric proteins, like HJURP and CENPA. Cells reduced in HJURP developed a higher proportion of misshapen, multilobed nuclei, or contained micronuclei (Foltz et al., 2009). These morphological abnormalities drive chromosome missegregation events underlying the interphase nuclear defects. HJURP-depleted cells also exhibited defects in chromosome segregation (Foltz et al., 2009). CENPA depletion also showed CIN phenotypes (Régnier et al., 2005).

**FIGURE 10:**
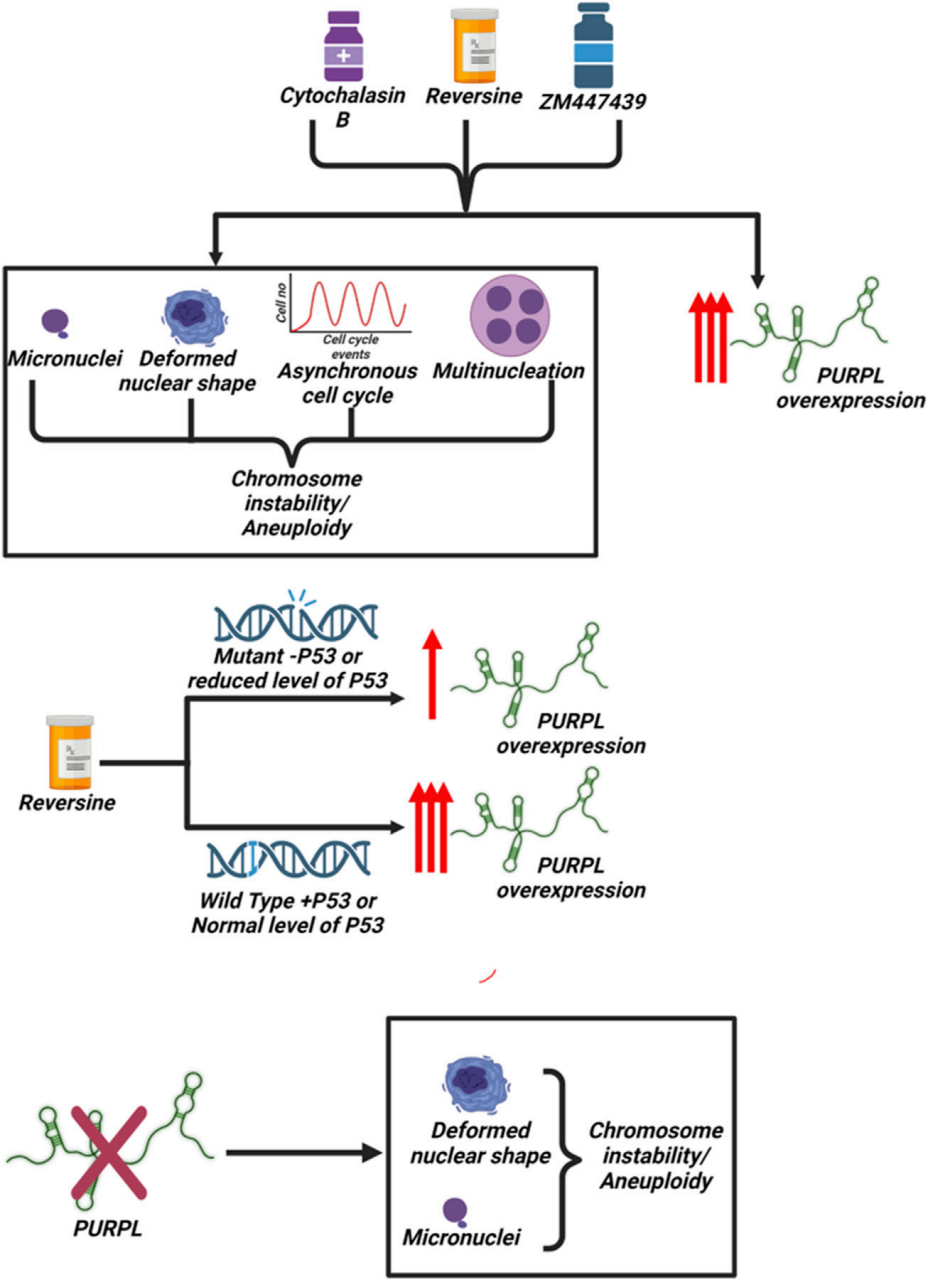
Scheme Summarizing the association of PURPL with chromosomal Instability/Aneuploidy: Treatment of cells with Reversine, Cytochalasin-B and ZM447439 resulted in induction of chromosomal instability/aneuploidy along with increased expression of long noncoding RNA PURPL. P53 determines the extent or magnitude of PURPL expression. Furthermore, knockdown of PURPL resulted in induction of chromosomal instability and aneuploidy.

In conclusion, our data demonstrated that PURPL is upregulated in reversine, cytochalasin B, and Aurora Kinase Inhibitor (ZM447439)-treated cells and is associated with chromosome instability and genomic stability (Figure 10). The present study indicates that PURPL can be a strong biomarker for chromosomal instability or genomic instability. Furthermore, the study clearly indicates that the expression of PURPL is not solely dependent on P53 status, though P53 determines the extent and magnitude of PURPL expression (Figure 10). Silencing PURPL promotes apoptosis. In addition, it affects chromosomal instability and affects nuclear morphology (Figure 10). Our future studies aim to investigate the specific mechanisms by which PURPL affects chromosomal instability or genomic instability. Collectively, lncRNA-PURPL was shown to exert an important contribution to CIN, or aneuploidy (Figure 10).

## Data Availability

The original contributions presented in the study are included in the article/Supplementary Material, further inquiries can be directed to the corresponding author.
