# Supplementary material for: Long noncoding RNA, PURPL is associated with aneuploidy and its magnitude of expression level is dependent on P53 status
Source: Front Cell Dev Biol. 2024 Dec 23;12:1410308. doi: 10.3389/fcell.2024.1410308 (PMC11702365; doi:10.3389/fcell.2024.1410308)
Supplement: Supplementary file 1 [file DataSheet1.PDF]

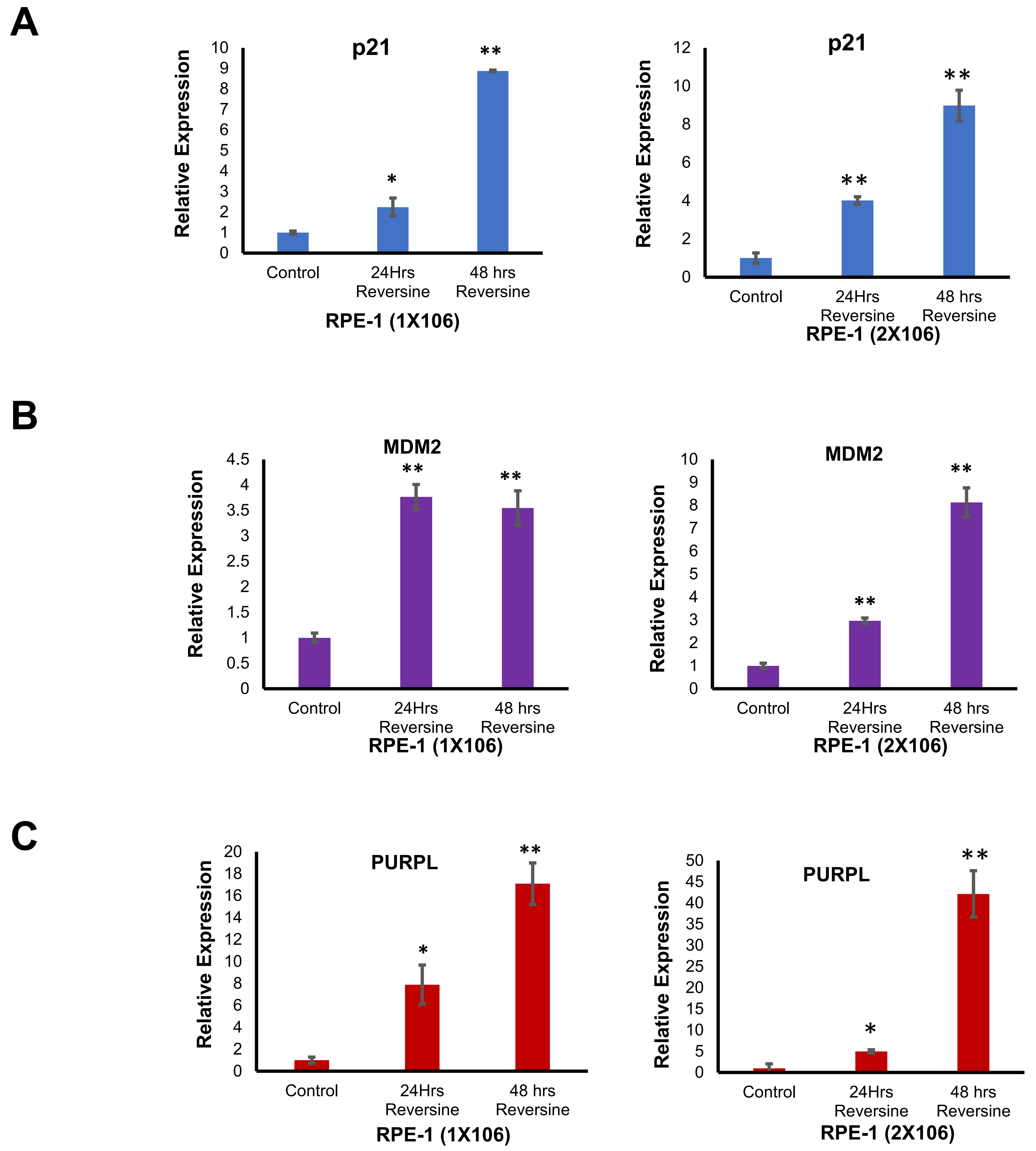

Reversine treatment leads to increased expression of the long noncoding RNA PURPL. RPE-1 cells were treated with reversine (1µM), and expression of PURPL was measured. **A).** qRT-PCR analysis showing p21 expression levels following reversine treatment. **B).** qRT-PCR analysis showing the expression levels of MDM2 upon reversine treatment. **C).** qRT-PCR analysis showing the expression levels of long noncoding RNA, PURPL, upon reversine treatment. Error bars, SD (n =≥2). Student t test was used. *, P < 0.05; **, P < 0.01; ***, P < 0.001.

FigureS1

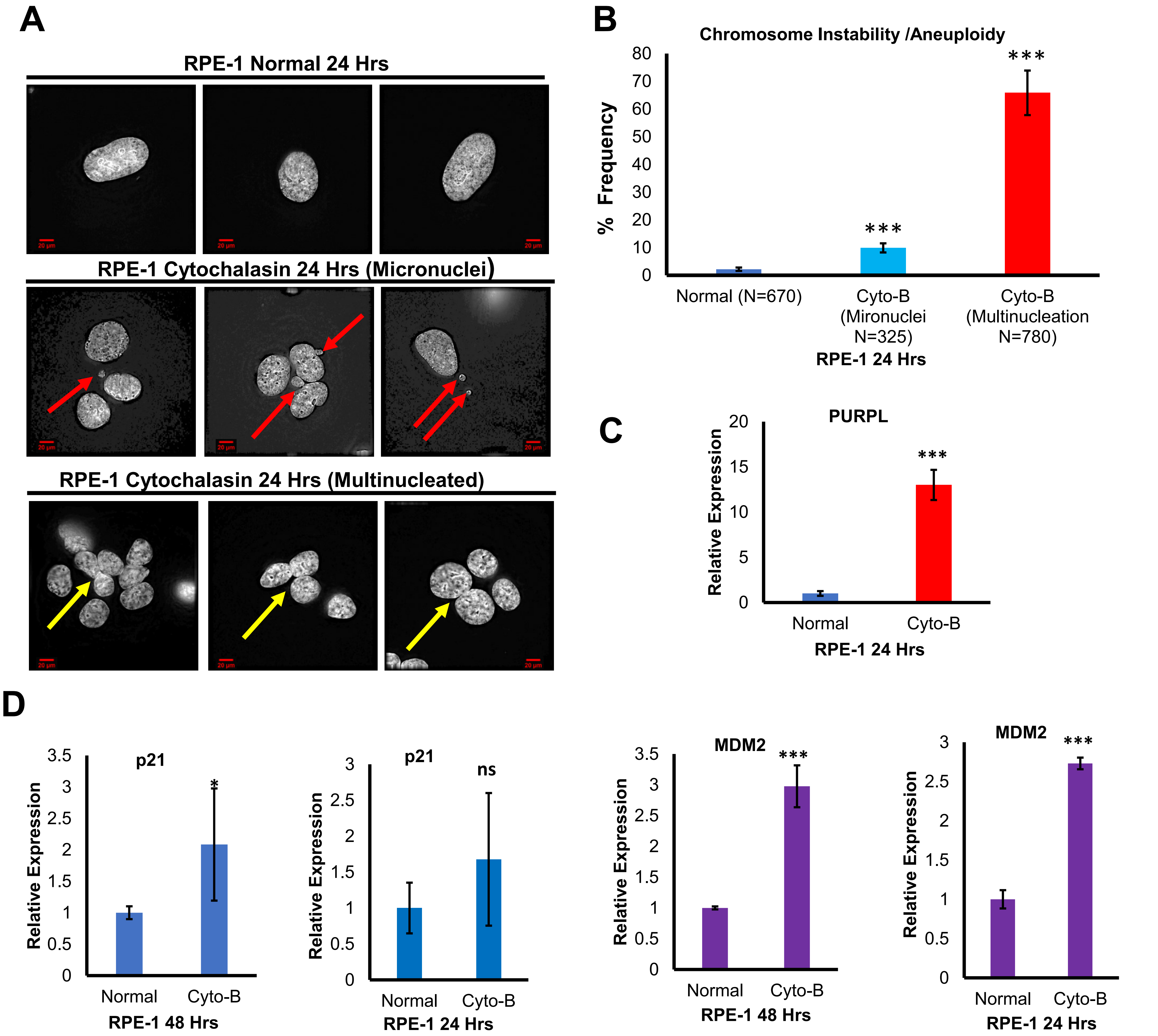

Cytochalasin-B treatment causes an increased incidence of micronuclei and multinucleation, as well as increased expression of the long noncoding RNA PURPL. **A).** RPE-1 cells were treated with Cytochalasin B (5 µM), and the incidence of micronuclei and multinucleation was measured after 24 hours. Cytochalasin B treatment contributes to an increased incidence of micronuclei and multinucleation in RPE-1 cells. Immunofluorescence images of the RPE-1 normal cells upper panel, the RPE-1 treated with Cytochalasin B middle panel showing the presence of micronuclei in interphase cells, and the RPE-1 treated with Cytochalasin B lower panel showing multinucleation. Red arrows indicate the presence of micronuclei. Yellow arrows show the presence of multinucleation. **B).** The graph on the right shows the quantification of micronuclei and multinucleation. **C).** qRT-PCR data analysis showing the expression levels of long noncoding RNA PURPL. **D).** qRT-PCR data analysis showing the expression levels of p21 and MDM2 in RPE-1 cells were treated with Cytochalasin B for 24 and 48 hours. Error bars, SD (n =≥2). Student t test was used. *, P < 0.05; **, P < 0.01; ***, P < 0.001.

FigureS2

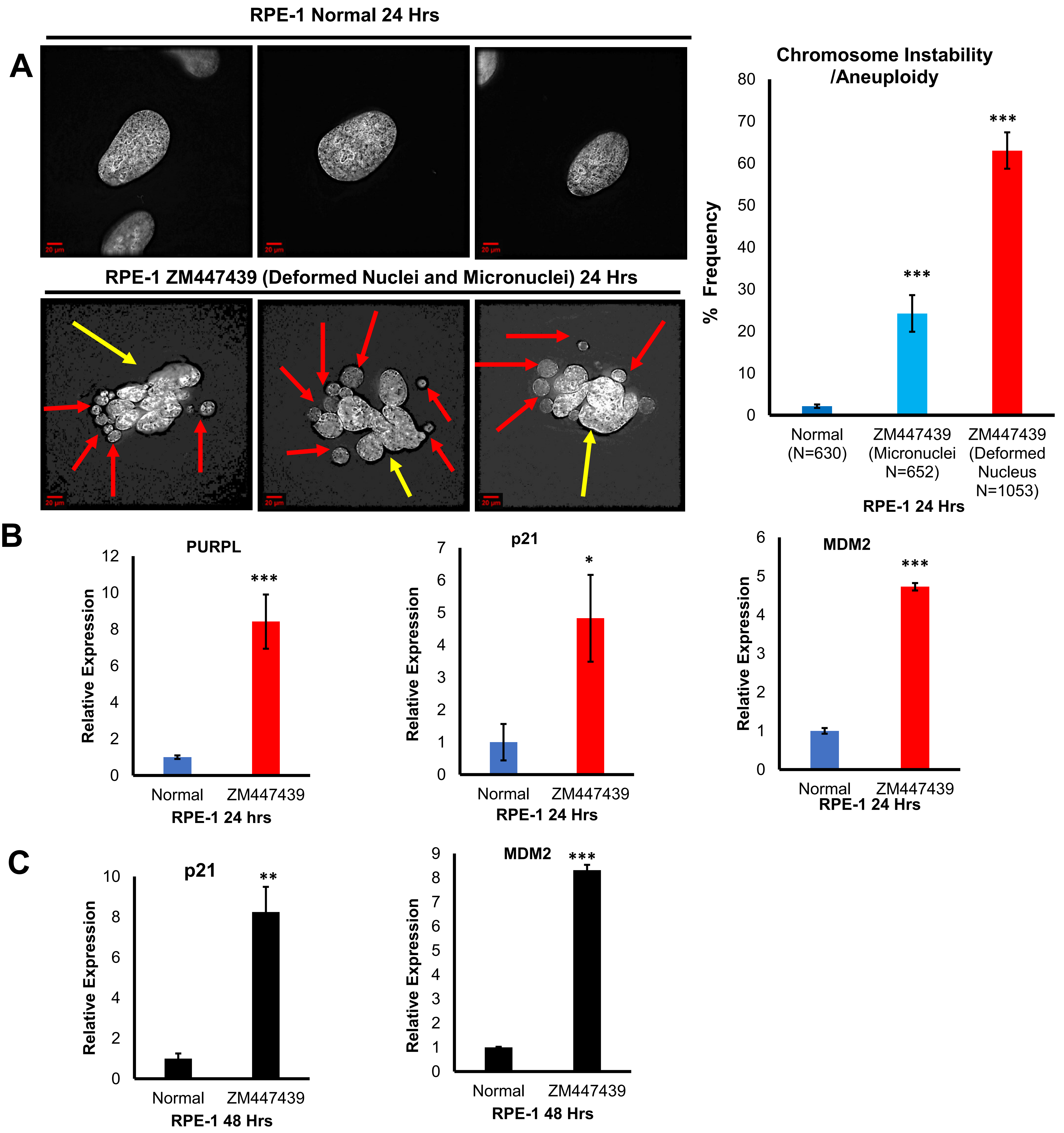

Aurora Kinase Inhibitor (ZM447439) treatment leads to increased incidence of micronuclei and deformed nuclei, as well as increased expression of the long noncoding RNA PURPL. A). RPE-1 cells were given ZM447439 (10 µM), and after 24 hours, the number of micronuclei and deformed nuclei was checked. ZM447439 treatment contributes to the increased incidence of micronuclei and deformed nuclei in RPE-1 cells. Immunofluorescence pictures of RPE-1 that has not been treated with ZM447439 (upper panel) and RPE-1 that has been treated with ZM447439 (lower panel) show that the nuclei are deformed and have micronuclei. Red arrows indicate the presence of micronuclei. Yellow arrows indicate the presence of a deformed nucleus. The graph on the right shows the quantification of micronuclei and deformed nuclei. B). qRT-PCR data analysis shows the expression levels of the long noncoding RNAs PURPL, p21, and MDM2. Increased expression of p21 and MDM2 are markers of p53 activation status during chromosomal instability. C). qRT-PCR data analysis showing the expression levels of p21 and MDM2 for RPE-1 cells treated with ZM447439 for 48 hours. Error bars, SD (n =≥2). A student t test was used. *, $P < 0.05$; **, $P < 0.01$; ***, $P < 0.001$.

FigureS3

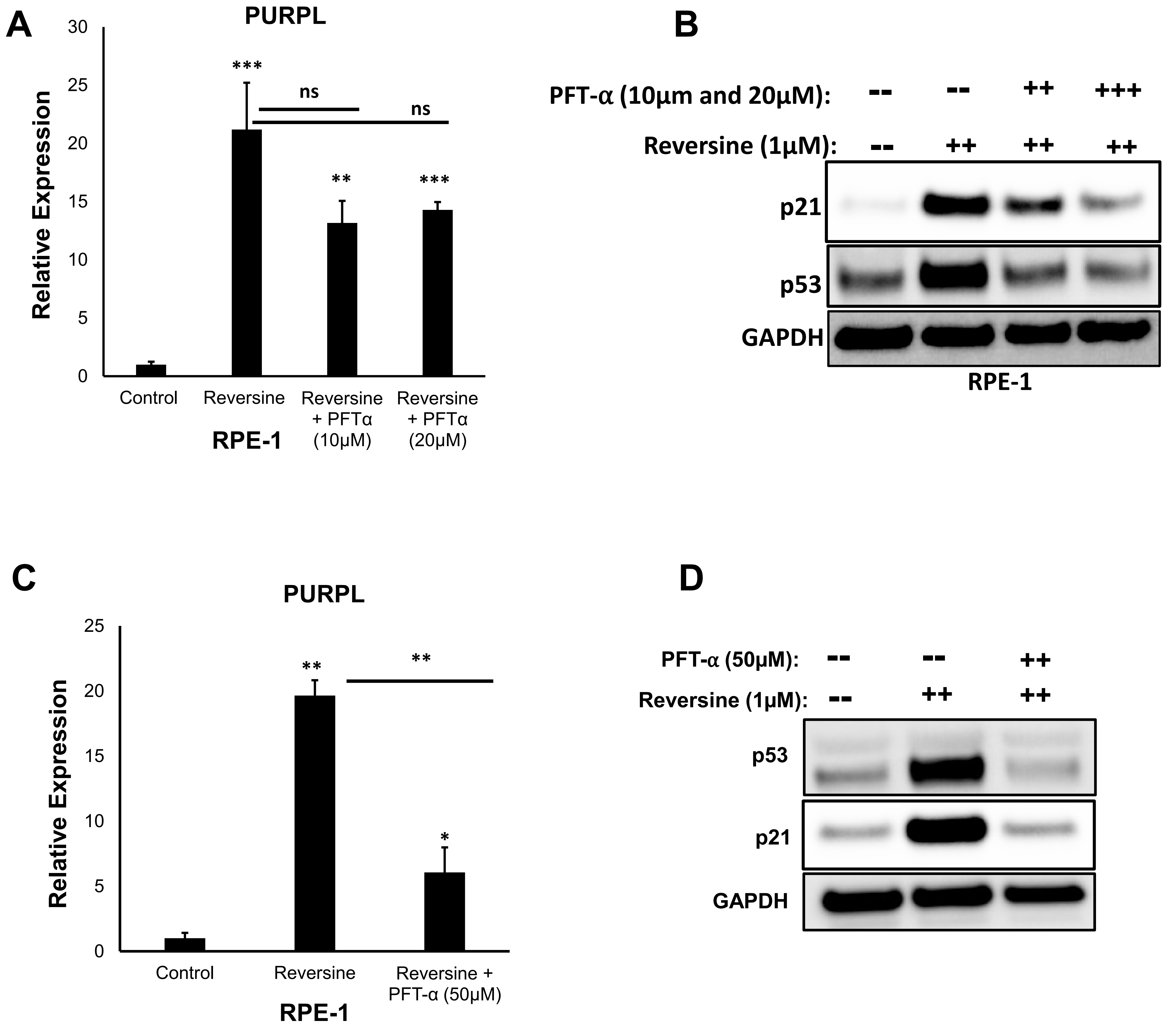

Reversine treatment in PFT-α resulted in reduced expression of the long noncoding RNA PURPL. A). *qRT-PCR analysis showing the expression levels of long noncoding RNA PURPL. The concentrations of PFT-α used were 10μM and 20μM.* ***B).*** *Western blot showing the levels of the indicated proteins. GAPDH was used as a loading control.* ***C).*** *qRT-PCR analysis shows the expression levels of the long noncoding RNA PURPL.* The concentration of PFT α used was 50 μM. ***D).*** *Western blot showing the levels of the indicated proteins. GAPDH was used as a loading control. The concentration of reversine used was 1 μM. The concentration of PFT α used was 50 μM. Error bars, SD (n =≥2). A student t-test was used. *, P < 0.05; **, P < 0.01; ***, P < 0.001.*

FigureS4

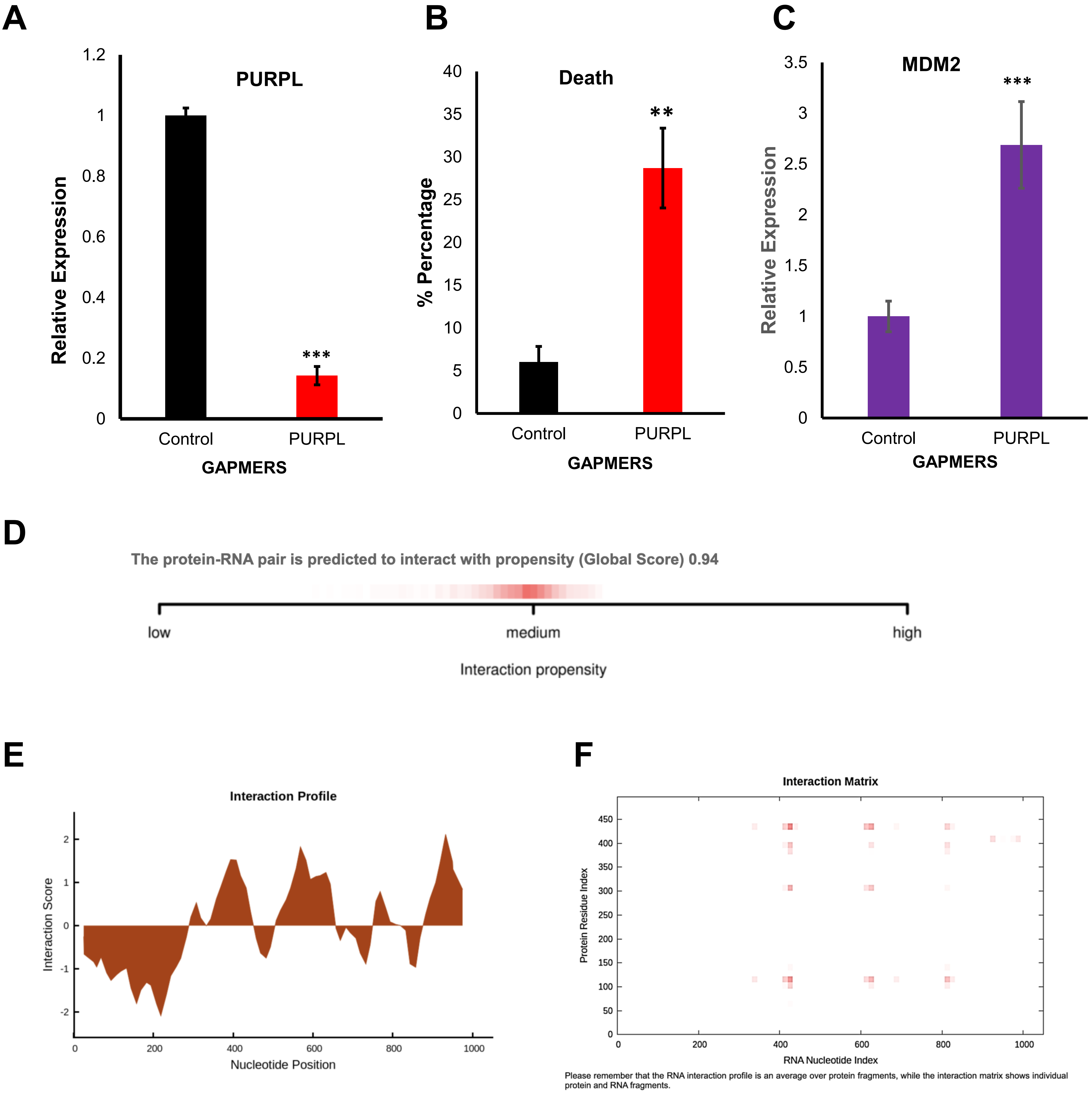

Long noncoding RNA PURPL knockdown contributes to cell death and increased expression of MDM2. A). qRT-PCR analysis showing the expression of PURPL in RPE-1 cells treated with GAPMERS either against PURPL or control. **B).** The cells described in (A) were analyzed by a trypan blue exclusion assay to determine the percentage of cell death. **C).** qRT-PCR showing the expression levels of MDM2. **D).** Interaction propensity of PURPL and MDM2 using catRAPID, a tool to predict protein-lncRNA interaction. **E).** Interaction profile of the nucleotide position of long noncoding RNA PURPL with MDM2. **F).** Interaction matrix involving long noncoding RNA PURPL nucleotide position and individual protein residue fragments of MDM2. Error bars, SD (n =≥2). A student t test was used. *, P < 0.05; **, P < 0.01; ***, P < 0.001.

Figure S5
